# Sequential Iron-Catalyzed C(sp^2^)–C(sp^3^) Cross-Coupling of Chlorobenzamides/Chemoselective Amide Reduction and Reductive Deuteration to Benzylic Alcohols

**DOI:** 10.3390/molecules28010223

**Published:** 2022-12-27

**Authors:** Elwira Bisz, Pamela Podchorodecka, Hengzhao Li, Wioletta Ochędzan-Siodłak, Jie An, Michal Szostak

**Affiliations:** 1 Department of Chemistry, Opole University, 48 Oleska Street, 45-052 Opole, Poland; 2 Department of Nutrition and Health, China Agricultural University, Beijing 100193, China; 3 Department of Chemistry, Rutgers University, 73 Warren Street, Newark, NJ 07102, USA

**Keywords:** sequential catalysis, amides, iron, sodium, cross-coupling, chemoselective reduction, N–C cleavage, iron catalysis, benzylic alcohols, deuterated compounds

## Abstract

Benzylic alcohols are among the most important intermediates in organic synthesis. Recently, the use of abundant metals has attracted significant attention due to the issues with the scarcity of platinum group metals. Herein, we report a sequential method for the synthesis of benzylic alcohols by a merger of iron catalyzed cross-coupling and highly chemoselective reduction of benzamides promoted by sodium dispersion in the presence of alcoholic donors. The method has been further extended to the synthesis of deuterated benzylic alcohols. The iron-catalyzed Kumada cross-coupling exploits the high stability of benzamide bonds, enabling challenging C(sp^2^)–C(sp^3^) cross-coupling with alkyl Grignard reagents that are prone to dimerization and β-hydride elimination. The subsequent sodium dispersion promoted reduction of carboxamides proceeds with full chemoselectivity for the C–N bond cleavage of the carbinolamine intermediate. The method provides access to valuable benzylic alcohols, including deuterium-labelled benzylic alcohols, which are widely used as synthetic intermediates and pharmacokinetic probes in organic synthesis and medicinal chemistry. The combination of two benign metals by complementary reaction mechanisms enables to exploit underexplored avenues for organic synthesis.

## 1. Introduction

Iron catalysis has found a major interest in organic synthesis owing to the issues associated with the limited supply and toxicity of platinum group metals [1,2,3,4,5,6,7,8,9,10,11,12,13,14,15,16,17,18,19,20,21,22,23,24]. The high abundance of iron as the most abundant transition metal in the Earth’s crust combined with the low biotoxicity is particularly attractive for the reaction development from the point of view of sustainability and global economy. Among cross-coupling catalysis, iron is one of the few metals that have found large scale industrial applications owing to the complementary reaction scope and compatibility to the palladium catalysis [25]. In this context, iron catalysis is a particularly attractive platform for the cross-coupling of alkyl Grignard reagents that feature β-hydrogens that are challenging using palladium catalysis [26,27,28].

Simultaneously, reduction of carboxamides represents one of the most important processes in organic synthesis [29,30,31,32]. This process uses amides as bench-stable precursors to afford downstream reduction products with high utility in medicinal chemistry, organic materials and agrochemistry [33,34,35]. Mechanistically, after the formation of the carbinolamine intermediate, C–O collapse leads to the formation of amine products, while C–N bond cleavage results in the formation of alcohols. In contrast to the typical metal hydrides, such as Al–H, B–H, which produce amines, amide reduction by C–N bond scission is much less common [36,37]. Furthermore, most reductants that lead to C–N collapse, give low chemoselectivity of the C–N scission [29,30,31,32,36,37], which is a major limitation considering a significant role of amides as bench-stable intermediates in organic synthesis. 

**Figure 1:**
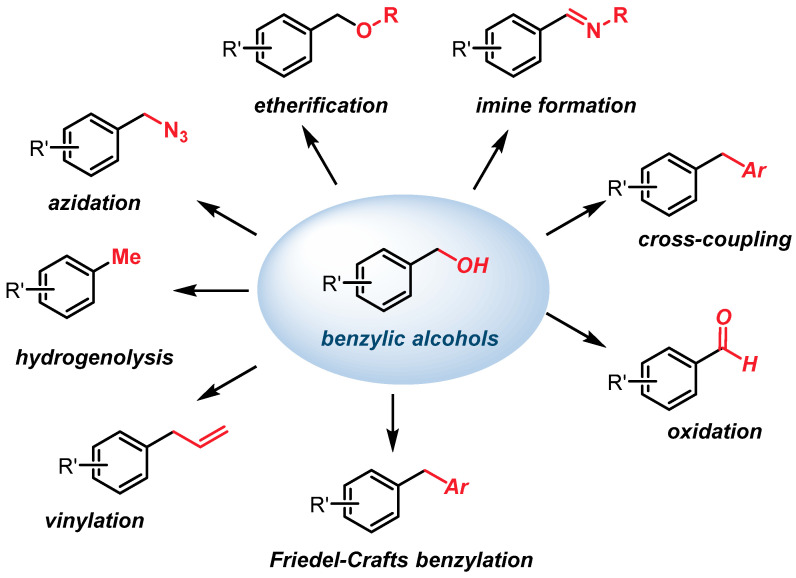
Synthetic applications of benzylic alcohols.

Benzylic alcohols are among the most important intermediates in organic synthesis. and valuable target compounds in their own right due to potent antimicrobial activity [33,34,35]. Selected synthetic applications of benzylic alcohols are presented in Figure 1. 

**Scheme 1:**
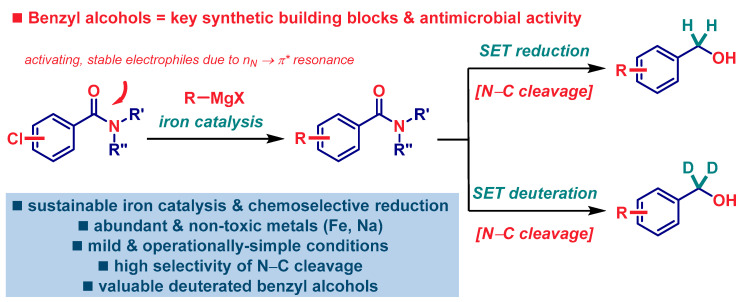
Sequential iron-catalyzed C(sp^2^)–C(sp^3^) Kumada cross-coupling of chlorobenzamides/chemoselective reduction and reductive deuteration to benzylic alcohols (this study).

Herein, we report a sequential method for the synthesis of benzylic alcohols by a merger of iron catalyzed cross-coupling and highly chemoselective reduction of benzamides promoted by sodium dispersion in the presence of alcoholic donors (Scheme 1). The following features of our study are noteworthy: (1) the iron-catalyzed Kumada cross-coupling exploits the high stability of benzamide bonds, enabling challenging C(sp^2^)–C(sp^3^) cross-coupling with alkyl Grignard reagents that are prone to dimerization and β-hydride elimination. (2) The subsequent sodium dispersion promoted reduction of carboxamides proceeds with full chemoselectivity for the C–N bond cleavage (cf. C–O) of the carbinolamine intermediate. (3) The method has been extended to the synthesis of deuterated benzylic alcohols with high deuterium incorporation (<90% D_2_). (4) The method is operationally simple, uses cheap, commercially available reagents and proton donors, and is performed with sustainable metals. Overall, the method provides access to valuable benzylic alcohols and deuterium-labelled benzylic alcohols, which are widely used as synthetic intermediates and pharmacokinetic probes in organic synthesis and medicinal chemistry. In a broader context, the combination of two abundant metals, Fe and Na, by complementary reaction mechanisms bodes significant potential for exploring new avenues in organic synthesis.

## 2. Results

As a part of our program in amide bonds [38,39,40,41] and iron catalysis [42,43,44,45,46,47,48,49,50,51,52], we considered a merger of the iron-catalyzed cross-coupling of amides with the subsequent chemoselective amide bond reduction. We hypothesized that the high stability of amide bonds would enable operationally simple access to the historically challenging C(sp^2^)–C(sp^3^) Kumada cross-coupling with alkyl Grignard reagents [26,27,28]. Furthermore, we became cognizant of the recent progress in chemoselective amide reduction by SET processes [53,54,55,56,57]. The method enables to exploit high stability of the N–C(O) amide bond by amidic resonance in iron-catalyzed cross-coupling, and chemoselectively tune the amide bond for SET reduction. The usage of sequential processes permits to generate value-added benzylic alcohols with high atom economy under sustainable and benign reaction conditions.

Our study commenced with an evaluation of the reaction conditions for the cross-coupling of a model 4-chloro-*N*,*N*-dimethylbenzamide with *n*-hexylmagnesium chloride. To date, the most synthetically useful system for iron-catalyzed cross-coupling has been established by Fürstner using NMP as an additive (NMP = N-methyl-2-pyrrolidone) [58,59,60,61,62,63,64,65,66,67]. However, due to mutagenicity of NMP and a major concern for the future use [68], several alternative and more benign promoters have been developed that feature similar arrangement of the *O*-coordination through N_lp_ → π* delocalization [1,2,3,4,5,6,7,8,9,10,11,12,13,14,15,16,17,18,19,20,21,22,23,24,42,43,44,45,46,47,48,49,50,51,52]. After experimentation, we found that although no reaction took place in the absence of iron catalyst (Table 1, entry 1) and the reaction was inefficient in the absence of additives (Table 1, entry 2), the addition of DMI (DMI = 1,3-dimethyl-2-imidazolidinone) resulted in 90% yield of the cross-coupling product (Table 1, entry 3). It is worthwhile to note that this reaction proceeded at low catalyst loading (0.10 mol%) in renewable 2-MeTHF as a solvent [69,70]. This solvent is slightly preferred over THF, most likely due to improved solubility of the reagents under these reaction conditions (Table 1, entry 4). Furthermore, we established that the cross-coupling is very facile, proceeding even at −78 °C. This is rare in iron-catalyzed cross-coupling and highlights the activating effect of the amide bond on cross-coupling. Finally, we determined that the yield could be further improved by changing the stoichiometry of the Grignard reagent, resulting in close to quantitative yield under these conditions (Table 1, entry 6).

With the optimized conditions in hand, we next evaluated the scope of this Kumada C(sp^2^)–C(sp^3^) cross-coupling (Table 2). With regard to the amide bond component, the reaction is very broad and accommodates various amides as activating groups. As such, cyclic amides, such as *N*-morpholinyl (**2a**), *N*-piperidinyl (**2b**), *N*-pyrrolidinyl (**2c**) and even highly strained *N*-azetidinyl (**2d**) are readily compatible (Table 2, entries 1–4). These examples demonstrated that the iron-catalyzed conditions can accommodate various amides, such as chelating *N*-morpholinyl (Table 1, entry 1) and reactive amides, such as *N*-azetidinyl (Table 1, entry 4), without addition of the Grignard reagent to the C(acyl)–N bond or cleavage of the alternative N–C bond. Furthermore, aliphatic amides with variable sterics, such as *N*,*N*-dimethyl (**2e**), *N*,*N*-diethyl (**2f**) and even highly hindered *N*,*N*-diisopropyl (**2g**) were compatible and afforded the corresponding products in high yields (Table 1, entries 5–7). Moreover, anilides featuring decreased amide N–C(O) conjugation due to N_lp_ delocalization onto the *N*-aromatic ring, such as **2h**, are compatible (Table 1, entry 8), attesting to the mild conditions of the present approach. Furthermore, benzylic amides also undergo cross-coupling in high yields (**2i**) (Table 1, entry 9), while the cleavage of the weak *N*–Bn bond is not observed under these mild iron-catalyzed conditions. Next, we briefly evaluated the scope of Grignard reagents. Importantly, we found that Grignard reagents featuring sterically demanding secondary substitution, such as cyclohexyl (**2j**) and isopropyl (**2k**) are compatible (Table 2, entries 10–11). The latter example is particularly noteworthy as the isomerization to the linear product was not observed, attesting to the fast cross-coupling vs. isomerization. Note that isomerization of secondary Grignard reagents is commonly observed using other iron-catalyzed cross-coupling methods, highlighting the mild nature of the present protocol. Finally, the reaction is also compatible with meta-chlorobenzamides (**2l**) (Table 2, entry 12). At present, the method is not compatible with ortho-chlorobenzamides, which are recovered unchanged due to the steric demand of the amide bond (not shown). At this stage, bulky Grignard reagents are not tolerated. An ongoing project is aimed at cross-coupling of bulky Grignards. At present, heteroaromatic substrates are not tolerated. An ongoing project addresses cross-coupling of heterocyclic substrates. These studies will be published in due course.

Having determined the scope of iron-catalyzed cross-coupling, we next moved on to establish the amide bond reduction as the second step of the sequential protocol. For this process, we selected the conditions using sodium dispersion and ethanol as a proton donor due to the high selectivity of amide bond cleavage, operational simplicity in the absence of pyrophoric metal hydrides and availability of the reagents [53,54,55,56,57]. Screening of the reaction conditions revealed that the ratio of sodium to EtOH of 1:3 is preferred (Table 3, entry 1), while the lower (Table 3, entry 2) or higher ratio (Table 3, entries 3–4) gave decreased yields. This reaction uses 4 equiv of sodium as a single electron donor. The reduction using close to a stoichiometric amount of sodium (Table 3, entry 5) is also feasible, albeit in lower yield. The optimized conditions give full selectivity for C–N/C–O cleavage.

The reduction proceeds via a SET mechanism with single electrons as reductants, and the first electron transfer as the rate determining step. Benzaldehyde is typically not detected in these reactions since its reduction is faster than amide reduction [53,54,55,56,57].

With the optimized reduction conditions in hand, we next evaluated the scope of the benzamide reduction using alkyl-benzamides prepared by the iron-catalyzed cross-coupling (Table 4). We found that this amide reduction is very general and accommodates various amide substrates in high yields. As shown, cyclic alkyl-benzamides, such as *N*-morpholinyl (**2a**), *N*-piperidinyl (**2b**), *N*-pyrrolidinyl (**2c**) and *N*-azetidinyl (**2d**) furnished the reduction products in 80-90% yields. Furthermore, aliphatic amides, such as *N*,*N*-dimethyl (**2e**), *N*,*N*-diethyl (**2f**) and *N*,*N*-diisopropyl (**2g**) were well-compatible, despite larger hindrance of the amide bond. Furthermore, anilides (**2h**) and *N*-benzylic amides (**2i**) can be successfully reduced. Finally, different substitution on the para (**2j–2k**) and meta position (**2l**) of the aromatic ring is compatible. Overall, this reduction processes tolerates a variety of substrates prepared by the iron-catalyzed cross-coupling, providing alkylated-benzylic alcohols with substantial utility as synthetic intermediates and antimicrobial agents [71].

Considering the recent interest in the synthesis of deuterium-labelled compounds as probes in pharmaceutical and agrochemical industry [72,73,74,75,76], we then became intrigued by the potential to extend the present sequential method to the synthesis of deuterated benzylic alcohols. The use of sodium dispersion in combination with a proton donor enables to readily incorporate deuterium label at the benzylic position. As shown in Table 5, these conditions are compatible with a range of alkyl-benzamides prepared by the iron-catalyzed cross-coupling to afford deuterated benzyl alcohols with > 90% deuterium incorporation. The yields obtained in the reductive deuteration are comparable with the efficiency of the reduction. As such, this process is equally effective for *N*-cyclic benzamides, *N*-morpholinyl (**2a**), *N*-piperidinyl (**2b**), *N*-pyrrolidinyl (**2c**) and *N*-azetidinyl (**2d**) as well as *N*-aliphatic benzamides, *N*,*N*-dimethyl (**2e**), *N*,*N*-diethyl (**2f**) and *N*,*N*-diisopropyl (**2g**), affording the products in 59–97% yields with 93–96% D_2_-incorporation. Similarly, anilides (**2h**), *N*-benzylic amides (**2i**) and different substitution (**2j–2l**) is tolerated, affording the products in 50–90% yields with 93–95% D_2_-incorporation. Overall, the reaction represents an operationally simple and cost-effective synthesis of deuterated benzylic alcohols, which are of interest as labelled probes. It is important to note that these reductions typically do not show significant isotope effect. SET is typically rate determining step [53,54,55,56,57].

**Scheme 2:**
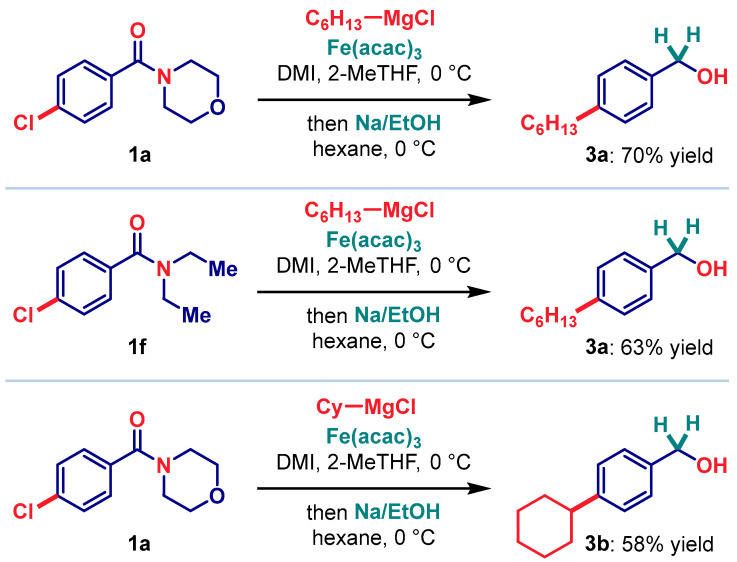
One-pot iron-catalyzed C(sp^2^)–C(sp^3^) Kumada cross-coupling/chemoselective reduction.

Finally, to demonstrate the utility of this iron-catalyzed cross-coupling/chemoselective amide reduction, we performed a one-pot sequential process (Scheme 2). As shown, the iron-catalyzed cross-coupling under standard conditions, followed by solvent exchange, and sodium dispersion mediated chemoselective reduction enables the synthesis of benzylic alcohols in the same pot. This reaction highlights the utility of completing the tandem cross-coupling/reduction process by combining two sustainable metals in a one-pot procedure. Work is currently in progress to develop in situ sequential processes involving iron-catalyzed cross-coupling as a key step. These studies will be published in due course.

## 3. Discussion

In summary, we have reported a sequential synthesis of benzylic alcohols by a merger of iron catalyzed cross-coupling and highly chemoselective reduction of benzamides promoted by sodium dispersion in the presence of alcoholic donors. Important aspects of this approach include iron-catalyzed Kumada cross-coupling that exploits the high stability of benzamide bonds, enabling challenging C(sp^2^)–C(sp^3^) cross-coupling with alkyl Grignard reagents that are prone to dimerization and β-hydride elimination and highly chemoselective, sodium dispersion promoted reduction of carboxamides that proceeds with full selectivity for the C–N bond cleavage. Moreover, this approach has been further extended to the synthesis of deuterated benzylic alcohols with high D_2_ incorporation. This study clearly indicates that the combination of abundant metals by complementary reaction mechanisms provides an attractive method for modular construction of important building blocks and pharmaceutical labels in organic synthesis. Our future studies are focused on developing sequential approaches for catalysis that would address the global issue of limited resources of transition metals.

## 4. Materials and Methods

**General Procedure for Iron-Catalyzed C(sp^2^)–C(sp^3^) Cross-Coupling of Chlorobenzamides.** An oven-dried vial equipped with a stir bar was charged with an amide substrate (neat, typically, 0.50 mmol, 1.0 equiv) and Fe(acac)_3_ (0.1 mol%), placed under a positive pressure of argon and subjected to three evacuation/backfilling cycles under vacuum. 2-Methyltetrahydrofuran (1.0 M) and DMI (neat, 200 mol%) were sequentially added with vigorous stirring at room temperature, the reaction mixture was cooled to 0 °C, a solution of Grignard reagent (typically, 1.05 equiv) was added dropwise with vigorous stirring and the reaction mixture was stirred for 18 h at 0 °C. After the indicated time, the reaction mixture was diluted with HCl (1.0 *N*, 1.0 mL) and EtOAc (1 × 30 mL), the organic layer was extracted with HCl (1.0 *N*, 2 × 10 mL), dried and concentrated. The sample was analyzed by ^1^H NMR (CDCl_3_, 400 MHz) to obtain conversion, yield and selectivity using internal standard and comparison with authentic samples. Analytical sample was purified by chromatography on silica gel (EtOAc/hexanes). Note that all reactions have been carried out using new glassware. Control reactions have been carried out using new glassware, stir bars, spatulas. It is worthwhile to note that palladium cannot easily catalyze the Kumada cross-coupling with alkyl Grignards due to fast β-hydride elimination [27,28]. All products are oils. Spectra are provided in the Supplementary Materials.

*Characterization Data of Cross-Coupling Products (Supplementary Materials)*

**(4-Hexylphenyl)(morpholino)methanone (Table 2, 2a).** Prepared according to the general procedure using (4-chlorophenyl)(morpholino)methanone (0.50 mmol), Fe(acac)_3_ (0.1 mol%), DMI (200 mol%), 2-MeTHF (1.0 M), and C_6_H_13_MgCl (2.0 M in THF, 1.05 equiv). The reaction mixture was stirred for 18 h at 0 °C. Yield 92% (126.5 mg). Colorless oil. ^1^H NMR (400 MHz, CDCl_3_) δ 7.32 (d, *J* = 8.2 Hz, 2H), 7.21 (d, *J* = 8.2 Hz, 2H), 3.93–3.36 (m, 8H), 2.62 (t, *J* = 7.7 Hz, 2H), 1.65–1.55 (m, 2H), 1.37–1.25 (m, 6H), 0.88 (t, *J* = 6.8 Hz, 3H). ^13^C NMR (100 MHz, CDCl_3_) δ 170.7, 145.1, 132.5, 128.5, 127.2, 77.4, 77.1, 76.8, 66.9, 48.3, 42.6, 35.8, 31.7, 31.3, 28.9, 22.6, 14.1. Spectroscopic properties matched those described previously [45].

**(4-Hexylphenyl)(piperidin-1-yl)methanone (Table 2, 2b).** Prepared according to the general procedure using (4-chlorophenyl)(piperidin-1-yl)methanone (0.50 mmol), Fe(acac)_3_ (0.1 mol%), DMI (200 mol%), 2-MeTHF (1.0 M), and C_6_H_13_MgCl (2.0 M in THF, 1.05 equiv). The reaction mixture was stirred for 18 h at 0 °C. Yield 92% (125.9 mg). Colorless oil. ^1^H NMR (400 MHz, CDCl_3_) δ 7.30 (d, *J* = 8.2 Hz, 2H), 7.19 (d, *J* = 8.3 Hz, 2H), 3.69 (brs, 2H), 3.36 (brs, 2H), 2.61 (t, *J* = 7.7 Hz, 2H), 1.71–1.44 (m, 8H), 1.37–1.24 (m, 6H), 0.88 (t, *J* = 6.7 Hz, 3H). ^13^C NMR (100 MHz, CDCl_3_) δ 170.6, 144.5, 133.7, 128.4, 126.9, 77.4, 77.1, 76.8, 48.8, 43.2, 35.8, 31.7, 31.3, 29.0, 26.6, 25.7, 24.7, 22.6, 14.1. Spectroscopic properties matched those described previously [45].

**(4-Hexylphenyl)(pyrrolidin-1-yl)methanone (Table 2, 2c).** Prepared according to the general procedure using (4-chlorophenyl)(pyrrolidin-1-yl)methanone (0.50 mmol), Fe(acac)_3_ (0.1 mol%), DMI (200 mol%), 2-MeTHF (1.0 M), and C_6_H_13_MgCl (2.0 M in THF, 1.20 equiv). The reaction mixture was stirred for 18 h at 0 °C. Yield 88% (114.1 mg). Colorless oil. ^1^H NMR (400 MHz, CDCl_3_) δ 7.44 (d, *J* = 8.2 Hz, 2H), 7.19 (d, *J* = 8.3 Hz, 2H), 3.64 (t, *J* = 7.0 Hz, 2H), 3.45 (t, *J* = 6.6 Hz, 2H), 2.62 (t, *J* = 7.7 Hz, 2H), 2.00–1.91 (m, 2H), 1.90–1.82 (m, 2H), 1.64–1.56 (m, 2H), 1.35–1.25 (m, 6H), 0.88 (t, *J* = 6.8 Hz, 3H). ^13^C NMR (100 MHz, CDCl_3_) δ 169.9, 144.9, 134.5, 128.2, 127.2, 77.2, 77.1, 76.7, 49.6, 46.2, 35.8, 31.7, 31.2, 28.9, 26.4, 24.4, 22.6, 14.1. Spectroscopic properties matched those described previously [77].

**Azetidin-1-yl(4-hexylphenyl)methanone (Table 2, 2d).** Prepared according to the general procedure using azetidin-1-yl(4-chlorophenyl)methanone (0.50 mmol), Fe(acac)_3_ (0.1 mol%), DMI (200 mol%), 2-MeTHF (1.0 M), and C_6_H_13_MgCl (2.0 M in THF, 1.05 equiv). The reaction mixture was stirred for 18 h at 0 °C. Yield 73% (89.6 mg). Colorless oil. *New compound*. ^1^H NMR (400 MHz, CDCl_3_) δ 7.55 (d, *J* = 8.3 Hz, 2H), 7.20 (d, *J* = 8.4 Hz, 2H), 4.31 (t, *J* = 7.5 Hz, 2H), 4.22 (t, *J* = 7.7 Hz, 2H), 2.62 (t, *J* = 7.7 Hz, 2H), 2.37–2.29 (m, 2H), 1.64–1.56 (m, 2H), 1.35–1.25 (m, 6H), 0.88 (t, *J* = 6.8 Hz, 3H). ^13^C NMR (100 MHz, CDCl_3_) δ 170.4, 146.1, 130.6, 128.3, 127.9, 77.4, 77.1, 76.7, 53.4, 48.9, 35.9, 31.7, 31.2, 28.9, 22.6, 16.1, 14.14. HRMS (ESI/Q-TOF) m/z: [M + Na]^+^ calcd for C_16_H_23_NONa 268.1677 found 268.1673.

**4-Hexyl-*N*,*N*-dimethylbenzamide (Table 2, 2e).** Prepared according to the general procedure using 4-chloro-*N*,*N*-dimethylbenzamide (0.50 mmol), Fe(acac)_3_ (0.1 mol%), DMI (200 mol%), 2-MeTHF (1.0 M), and C_6_H_13_MgCl (2.0 M in THF, 1.05 equiv). The reaction mixture was stirred for 18 h at 0 °C. Yield 95% (110.9 mg). Colorless oil. ^1^H NMR (400 MHz, CDCl_3_) δ 7.33 (d, *J* = 8.1 Hz, 2H), 7.19 (d, *J* = 8.1 Hz, 2H), 3.10 (brs, 3H), 2.99 (brs, 3H), 2.61 (t, *J* = 7.7 Hz, 2H), 1.65–1.55 (m, 2H), 1.37–1.25 (m, 6H), 0.88 (t, *J* = 6.8 Hz, 3H). ^13^C NMR (100 MHz, CDCl_3_) δ 171.8, 144.5, 133.5, 128.2, 127.1, 77.4, 77.1, 76.7, 39.6, 35.7, 35.3, 31.6, 31.2, 28.8, 22.5, 14.0. Spectroscopic properties matched those described previously [45].

***N*,*N*-Diethyl-4-hexylbenzamide (Table 2, 2f).** Prepared according to the general procedure using 4-chloro-N,N-diethylbenzamide (0.50 mmol), Fe(acac)_3_ (0.1 mol%), DMI (200 mol%), 2-MeTHF (1.0 M), and C_6_H_13_MgCl (2.0 M in THF, 1.20 equiv). The reaction mixture was stirred for 18 h at 0 °C. Yield 98% (128.3 mg). Colorless oil. *New compound*. ^1^H NMR (400 MHz, CDCl_3_) δ 7.28 (d, *J* = 8.1 Hz, 2H), 7.19 (d, *J* = 8.2 Hz, 2H), 3.54 (brs, 2H), 3.28 (brs, 2H), 2.61 (t, *J* = 7.7 Hz, 2H), 1.65–1.55 (m, 2H), 1.36–1.06 (m, 12H), 0.88 (t, *J* = 6.8 Hz, 3H). ^13^C NMR (100 MHz, CDCl_3_) δ 171.5, 144.1, 134.5, 128.3, 126.3, 77.4, 77.1, 76.7, 43.3, 39.2, 35.8, 31.7, 31.3, 28.9, 22.6, 14.2, 14.1, 12.9. HRMS (ESI/Q-TOF) m/z: [M + Na]^+^ calcd for C_17_H_27_NONa 284.1990 found 284.1985.

**4-Hexyl-*N*,*N*-diisopropylbenzamide (Table 2, 2g).** Prepared according to the general procedure using 4-chloro-*N*,*N*-diisopropylbenzamide (0.50 mmol), Fe(acac)_3_ (0.1 mol%), DMI (200 mol%), 2-MeTHF (1.0 M), and C_6_H_13_MgCl (2.0 M in THF, 1.20 equiv). The reaction mixture was stirred for 18 h at 0 °C. Yield 98% (141.7 mg). Colorless oil. *New compound*. ^1^H NMR (400 MHz, CDCl_3_) δ 7.22 (d, *J* = 8.2 Hz, 2H), 7.17 (d, *J* = 8.3 Hz, 2H), 4.11–3.27 (m, 2H), 2.60 (t, *J* = 7.7 Hz, 2H), 1.65–1.07 (m, 20H), 0.88 (t, *J* = 6.8 Hz, 3H). ^13^C NMR (100 MHz, CDCl_3_) δ 171.3, 143.6, 136.2, 128.4, 125.7, 50.8, 45.8, 35.8, 31.7, 31.3, 28.9, 22.6, 20.8, 14.1. HRMS (ESI/Q-TOF) m/z: [M + Na]^+^ calcd for C_19_H_31_NONa 312.2303 found 312.2295.

**4-Hexyl-*N*-methyl-*N*-phenylbenzamide (Table 2, 2h).** Prepared according to the general procedure using 4-chloro-N-methyl-N-phenylbenzamide (0.50 mmol), Fe(acac)_3_ (0.1 mol%), DMI (200 mol%), 2-MeTHF (1.0 M), and C_6_H_13_MgCl (2.0 M in THF, 1.05 equiv). The reaction mixture was stirred for 18 h at 0 °C. Yield 85% (125.7 mg). Colorless oil. *New compound*. ^1^H NMR (400 MHz, CDCl3) δ 7.24–7.18 (m, 4H), 7.15–7.09 (m, 1H), 7.06–7.01 (m, 2H), 6.95 (d, *J* = 8.3 Hz, 2H), 3.49 (s, 3H), 2.49 (t, *J* = 7.7 Hz, 2H), 1.55–1.46 (m, 2H), 1.28–1.20 (m, 6H), 0.85 (t, *J* = 6.9 Hz, 3H). ^13^C NMR (100 MHz, CDCl_3_) δ 170.7, 145.1, 144.7, 133.0, 129.0, 128.8, 127.6, 126.8, 126.2, 77.4, 77.1, 76.7, 38.4, 35.6, 31.5, 30.9, 28.7, 22.5, 14.0. HRMS (ESI/Q-TOF) m/z: [M + Na]^+^ calcd for C_20_H_25_NONa 318.1834 found 318.1825.

***N*-Benzyl-4-hexyl-*N*-methylbenzamide (Table 2, 2i).** Prepared according to the general procedure using *N*-benzyl-4-chloro-N-methylbenzamide (0.50 mmol), Fe(acac)_3_ (0.1 mol%), DMI (200 mol%), 2-MeTHF (1.0 M), and C_6_H_13_MgCl (2.0 M in THF, 1.05 equiv). The reaction mixture was stirred for 18 h at 0 °C. Yield 95% (147.2 mg). Colorless oil. *New compound*. ^1^H NMR (400 MHz, CDCl3) δ 7.42–7.13 (m, 9H), 4.75 (brs, 1H), 4.54 (brs, 1H), 3.10–2.80 (m, 3H), 2.60 (brs, 2H), 1.59 (brs, 2H), 1.29 (brs, 6H), 0.87 (t, *J* = 6.8 Hz, 3H). ^13^C NMR (100 MHz, CDCl_3_) (mixture of two rotamers) δ 172.6, 171.8, 144.7, 137.1, 136.7, 133.3, 128.7, 128.4, 128.1, 127.5, 127.1, 126.9, 126.7, 77.4, 77.1, 76.7, 55.2, 50.8, 37.1, 35.8, 33.2, 31.7, 31.2, 28.9, 22.6, 14.1. HRMS (ESI/Q-TOF) m/z: [M + Na]^+^ calcd for C_21_H_27_NONa 332.1990 found 332.1975.

**(4-Cyclohexylphenyl)(morpholino)methanone (Table 2, 2j).** Prepared according to the general procedure using (4-chlorophenyl)(morpholino)methanone (0.50 mmol), Fe(acac)_3_ (0.1 mol%), DMI (200 mol%), 2-MeTHF (1.0 M), and *c*-C_6_H_11_MgCl (1.0 M in 2-MeTHF, 2.00 equiv). The reaction mixture was stirred for 18 h at 0 °C. Yield 80% (109.5 mg). White solid. ^1^H NMR (400 MHz, CDCl3) δ 7.33 (d, *J* = 8.3 Hz, 2H), 7.24 (d, *J* = 8.0 Hz, 2H), 3.89–3.40 (m, 8H), 2.58–2.46 (m, 1H), 1.92–1.80 (m, 4H), 1.80–1.71 (m, 1H), 1.48–1.32 (m, 4H), 1.32–1.17 (m, 1H). ^13^C NMR (100 MHz, CDCl_3_) δ 170.6, 150.1, 132.6, 127.2, 127.0, 77.4, 77.1, 76.7, 66.9, 48.4, 44.4, 42.6, 34.2, 26.7, 26.0. Spectroscopic properties matched those described previously [45].

**(4-Isopropylphenyl)(morpholino)methanone (Table 2, 2k).** Prepared according to the general procedure using (4-chlorophenyl)(morpholino)methanone (0.50 mmol), Fe(acac)_3_ (0.1 mol%), DMI (200 mol%), 2-MeTHF (1.0 M), and *i*-PrMgBr (0.6 M in THF, 2.0 equiv). The reaction mixture was stirred for 18 h at 0 °C. Yield 56% (65.4 mg). Colorless oil. ^1^H NMR (400 MHz, CDCl_3_) δ 7.34 (d, *J* = 8.3 Hz, 2H), 7.26 (d, *J* = 8.0 Hz, 2H), 3.85–3.44 (m, 8H), 2.99–2.85 (m, 1H), 1.25 (d, *J* = 6.9 Hz, 6H). ^13^C NMR (100 MHz, CDCl_3_) δ 170.7, 151.0, 132.7, 127.3, 126.6, 77.4, 77.1, 76.7, 66.9, 34.1, 23.8. Spectroscopic properties matched those described previously [78].

**(3-Hexylphenyl)(morpholino)methanone (Table 2, 2l).** Prepared according to the general procedure using (3-chlorophenyl)(morpholino)methanone (0.50 mmol), Fe(acac)_3_ (0.1 mol%), DMI (200 mol%), 2-MeTHF (1.0 M), and C_6_H_13_MgCl (2.0 M in THF, 1.05 equiv). The reaction mixture was stirred for 18 h at 0 °C. Yield 68% (93.5 mg). Colorless oil. *New compound*. ^1^H NMR(400 MHz, CDCl3) δ 7.34–7.28 (m, 1H), 7.25–7.16 (m, 3H), 3.90–3.37 (m, 8H), 2.62 (t, *J* = 7.7 Hz, 2H), 1.65–1.55 (m, 2H), 1.35–1.24 (m, 6H), 0.88 (t, *J* = 6.9 Hz, 3H). ^13^C NMR (101 MHz, CDCl_3_) δ 170.7, 143.4, 135.2, 129.9, 128.3, 127.0, 124.2, 77.4, 77.1, 76.7, 66.9, 48.1, 42.5, 35.7, 31.6, 31.2, 28.9, 22.5, 14.0. HRMS (ESI/Q-TOF) m/z: [M + Na]^+^ calcd for C_17_H_25_NO_2_Na 298.1783 found 298.1777.

Optimization Studies for the Reduction of Amides. To a solution of (4-cyclohexylphenyl)(morpholino)methanone (0.30 mmol) in solvent (2.5 mL), EtOH (4.5–15 mmol) was added, followed by sodium dispersions in oil (1.5–3.0 mmol) under Ar at 0 °C and the resulting solution was stirred vigorously. After 20 min the reaction mixture was quenched by an aqueous solution of NaHCO_3_ (5.0 mL, saturated) and the reaction mixture was diluted with EtOAc (10 mL) and brine (10 mL). The aqueous layer was extracted with EtOAc (2 × 10 mL), the organic layers were combined, dried over MgSO_4_, filtered and concentrated. Then, the sample was analyzed by ^1^H NMR (CDCl_3_, 300 MHz) to obtain the deuterium incorporation and yield using internal standard and comparison with authentic samples.

**General Procedure for the Reduction of Amides by Na/EtOH.** To a solution of amide substrate (0.30 mmol) in hexane (2.5 mL), EtOH (9.0 mmol) was added, followed by sodium dispersions in oil (34 wt%, 3.0 mmol) under Ar at 0 °C and the resulting solution was stirred vigorously. After 20 min, the reaction mixture was quenched by an aqueous solution of NaHCO_3_ (5.0 mL, saturated) and the reaction mixture was diluted with EtOAc (10 mL) and brine (10 mL). The aqueous layer was extracted with EtOAc (2 × 10 mL), the organic layers were combined, dried over MgSO_4_, filtered and concentrated. The crude product was purified by flash chromatography on silica gel (EtOAc/petroleum ether).

*Characterization Data of Reduction Products (Supplementary Materials)*

**(4-Hexylphenyl)methanol (**Table 4**, 3a).** According to the general procedure, the reaction of (4-hexylphenyl)(morpholino)methanone (0.30 mmol), EtOH (9.0 mmol) and Na dispersion in oil (3.0 mmol), after chromatography (0–25% EtOAc/petroleum ether), afforded **3a**, 51.9 mg, 90% yield as a colorless oil. ^1^H NMR (300 MHz, CDCl_3_) δ 7.27 (d, *J* = 7.9 Hz, 2H), 7.17 (d, *J* = 7.9 Hz, 2H), 4.62 (s, 2H), 2.59 (t, *J* = 7.7 Hz, 2H), 1.89 (br, 1H), 1.60 (m, 2H), 1.37–1.23 (m, 6H), 0.88 (t, *J* = 6.8 Hz, 3H); ^13^C NMR (75 MHz, CDCl_3_) δ 142.6, 138.2, 128.7, 127.2, 65.4, 35.8, 31.8, 31.6, 29.1, 22.7, 14.2. Spectroscopic properties matched those described previously [79].

**(4-Hexylphenyl)methanol (**Table 4**, 3a).** According to the general procedure, the reaction of (4-hexylphenyl)(piperidin-1-yl)methanone (0.30 mmol), EtOH (9.0 mmol) and Na dispersion in oil (3.0 mmol), after chromatography (0–25% EtOAc/petroleum ether), afforded **3a**, 46.7 mg, 81% yield as a colorless oil. Spectroscopic properties matched those described previously [79].

**(4-Hexylphenyl)methanol (**Table 4**, 3a).** According to the general procedure, the reaction of (4-hexylphenyl)(pyrrolidin-1-yl)methanone (0.30 mmol), EtOH (9.0 mmol) and Na dispersion in oil (3.0 mmol), after chromatography (0–25% EtOAc/petroleum ether), afforded **3a**, 51.9 mg, 90% yield as a colorless oil. Spectroscopic properties matched those described previously [79].

**(4-Hexylphenyl)methanol (**Table 4**, 3a).** According to the general procedure, the reaction of azetidin-1-yl(4-hexylphenyl)methanone (0.30 mmol), EtOH (9.0 mmol) and Na dispersion in oil (3.0 mmol), after chromatography (0–25% EtOAc/petroleum ether), afforded **3a**, 46.2 mg, 80% yield as a colorless oil. Spectroscopic properties matched those described previously [79].

**(4-Hexylphenyl)methanol (**Table 4**, 3a).** According to the general procedure, the reaction of 4-hexyl-*N,N*-dimethylbenzamide (0.30 mmol), EtOH (9.0 mmol) and Na dispersion in oil (3.0 mmol), after chromatography (0–25% EtOAc/petroleum ether), afforded **3a**, 43.3 mg, 75% yield as a colorless oil. Spectroscopic properties matched those described previously [79].

**(4-Hexylphenyl)methanol (**Table 4**, 3a).** According to the general procedure, the reaction of *N,N*-diethyl-4-hexylbenzamide (0.30 mmol), EtOH (9.0 mmol) and Na dispersion in oil (3.0 mmol), after chromatography (0–25% EtOAc/petroleum ether), afforded **3a**, 42.1 mg, 73% yield as a colorless oil. Spectroscopic properties matched those described previously [79].

**(4-Hexylphenyl)methanol (**Table 4**, 3a).** According to the general procedure, the reaction of 4-hexyl-*N,N*-diisopropylbenzamide (0.30 mmol), EtOH (9.0 mmol) and Na dispersion in oil (3.0 mmol), after chromatography (0–25% EtOAc/petroleum ether), afforded **3a**, 42.2 mg, 73% yield as a colorless oil. Spectroscopic properties matched those described previously [79].

**(4-Hexylphenyl)methanol (**Table 4**, 3a).** According to the general procedure, the reaction of 4-hexyl-*N*-methyl-*N*-phenylbenzamide (0.30 mmol), EtOH (9.0 mmol) and Na dispersion in oil (3.0 mmol), after chromatography (0–25% EtOAc/petroleum ether), afforded **3a**, 46.7 mg, 81% yield as a colorless oil. Spectroscopic properties matched those described previously [79].

**(4-Hexylphenyl)methanol (**Table 4**, 3a).** According to the general procedure, the reaction of *N*-benzyl-4-hexyl-*N*-methylbenzamide (0.30 mmol), EtOH (9.0 mmol) and Na dispersion in oil (3.0 mmol), after chromatography (0–25% EtOAc/petroleum ether), afforded **3a**, 36.9 mg, 64% yield as a colorless oil. Spectroscopic properties matched those described previously [79].

**(4-Cyclohexylphenyl)methanol (**Table 4**, 3b).** According to the general procedure, the reaction of (4-cyclohexylphenyl)(morpholino)methanone (0.30 mmol), EtOH (9.0 mmol) and Na dispersion in oil (3.0 mmol), after chromatography (0–25% EtOAc/petroleum ether), afforded **3b**, 49.7 mg, 87% yield as a white solid. ^1^H NMR (300 MHz, CDCl_3_) δ 7.29 (m, 2H), 7.20 (m, 2H), 4.64 (s, 2H), 2.49 (m, 1H), 1.93–1.60 (m, 7H), 1.49–1.33 (m, 4H); ^13^C NMR (75 MHz, CDCl_3_) δ 147.8, 138.4, 127.2, 127.1, 65.4, 44.4, 34.6, 27.0, 26.3. Spectroscopic properties matched those described previously [80].

**(4-Isopropylphenyl)methanol (**Table 4**, 3c).** According to the general procedure, the reaction of (4-isopropylphenyl)(morpholino)methanone (0.30 mmol), EtOH (9.0 mmol) and Na dispersion in oil (3.0 mmol), after chromatography (0–25% EtOAc/petroleum ether), afforded **3c**, 33.8 mg, 75% yield as a white solid. ^1^H NMR (300 MHz, CDCl_3_) δ 7.29 (m, 2H), 7.22 (m, 2H), 4.62 (s, 2H), 2.90 (m, 1H), 1.98 (br, 1H), 1.24 (d, *J* = 6.9 Hz, 6H); ^13^C NMR (75 MHz, CDCl_3_) δ 148.5, 138.4, 127.3, 126.7, 65.3, 34.0, 24.1. Spectroscopic properties matched those described previously [81].

**(3-Hexylphenyl)methanol (**Table 4**, 3d).** According to the general procedure, the reaction of (3-hexylphenyl)(morpholino)methanone (0.30 mmol), EtOH (9.0 mmol) and Na dispersion in oil (3.0 mmol), after chromatography (0–25% EtOAc/petroleum ether), afforded **3d**, 30.6 mg, 53% yield as a colorless oil. *New compound*. ^1^H NMR (400 MHz, CDCl_3_) δ 7.30–7.25 (m, 1H), 7.21–7.15 (m, 2H), 7.14–7.10 (m, 2H), 4.67 (s, 2H), 2.61 (t, *J* = 7.7 Hz, 2H), 1.69–1.57 (m, 3H), 1.35–1.25 (m, 6H), 0.88 (t, *J* = 6.8 Hz, 3H). ^13^C NMR (100 MHz, CDCl_3_) δ 143.4, 140.8, 128.5, 127.8, 127.1, 124.3, 77.4, 77.1, 76.7, 65.5, 36.0, 31.8, 31.5, 29.1, 22.6, 14.1. HRMS (ESI/Q-TOF) m/z: [M + Na]^+^ calcd for C_13_H_20_ONa 215.1412 found 215.1403.

**General Procedure for the Reductive Deuteration of Amides by Na/EtOD-*d_1_*.** To a solution of an amide substrate (0.30 mmol) in hexane (2.5 mL), EtOH (9.0 mmol) was added, followed by sodium dispersions in oil (34 wt%, 3.0 mmol) under Ar at 0 °C and the resulting solution was stirred vigorously. After 20 min, the reaction mixture was quenched by an aqueous solution of NaHCO_3_ (5.0 mL, saturated) and the reaction mixture was diluted with EtOAc (10 mL) and brine (10 mL). The aqueous layer was extracted with EtOAc (2 × 10 mL), the organic layers were combined, dried over MgSO_4_, filtered and concentrated. The crude product was purified by flash chromatography on slica gel (EtOAc/petroleum ether).

*Characterization Data of Reductive Deuteration Products (Supplementary Materials)*

**(4-Hexylphenyl)methan-*d_2_*-ol (Table 5, 4a)**. According to the general procedure, the reaction of (4-hexylphenyl)(morpholino)methanone (0.30 mmol), EtOD-*d_1_* (9.0 mmol) and Na dispersion in oil (3.0 mmol), after chromatography (0–25% EtOAc/petroleum ether), afforded **4a**, 44.3 mg, 76% yield as a colorless oil. ^1^H NMR (300 MHz, CDCl_3_) δ 7.26 (d, *J* = 8.1 Hz, 2H), 7.17 (d, *J* = 8.1 Hz, 2H), 2.60 (t, *J* = 7.7 Hz, 2H), 1.67 (br, 1H), 1.60 (m, 2H), 1.37–1.25 (m, 6H), 0.88 (t, *J* = 6.9 Hz, 3H); ^13^C NMR (75 MHz, CDCl_3_) δ 142.6, 138.2 (m), 128.7, 127.2, 65.0 (m), 35.8, 31.8, 31.6, 29.1, 22.7, 14.2. Spectroscopic properties matched those described previously [79].

**(4-Hexylphenyl)methan-*d_2_*-ol (Table 5, 4a)**. According to the general procedure, the reaction of (4-hexylphenyl)(piperidin-1-yl)methanone (0.30 mmol), EtOD-*d_1_* (9.0 mmol) and Na dispersion in oil (3.0 mmol), after chromatography (0–25% EtOAc/petroleum ether), afforded **4a**, 56.5 mg, 97% yield as a colorless oil. Spectroscopic properties matched those described previously [79].

**(4-Hexylphenyl)methan-*d_2_*-ol (Table 5, 4a).** According to the general procedure, the reaction of (4-hexylphenyl)(pyrrolidin-1-yl)methanone (0.30 mmol), EtOD-*d_1_* (9.0 mmol) and Na dispersion in oil (3.0 mmol), after chromatography (0–25% EtOAc/petroleum ether), afforded **4a**, 50.7 mg, 87% yield as a colorless oil. Spectroscopic properties matched those described previously [79].

**(4-Hexylphenyl)methan-*d_2_*-ol (Table 5, 4a).** According to the general procedure, the reaction of azetidin-1-yl(4-hexylphenyl)methanone (0.30 mmol), EtOD-*d_1_* (9.0 mmol) and Na dispersion in oil (3.0 mmol), after chromatography (0–25% EtOAc/petroleum ether), afforded **4a**, 42.6 mg, 73% yield as a colorless oil. Spectroscopic properties matched those described previously [79].

**(4-Hexylphenyl)methan-*d_2_*-ol (Table 5, 4a).** According to the general procedure, the reaction of 4-hexyl-*N,N*-dimethylbenzamide (0.30 mmol), EtOD-*d_1_* (9.0 mmol) and Na dispersion in oil (3.0 mmol), after chromatography (0–25% EtOAc/petroleum ether), afforded **4a**, 42.0 mg, 72% yield as a colorless oil. Spectroscopic properties matched those described previously [79].

**(4-Hexylphenyl)methan-*d_2_*-ol (Table 5, 4a).** According to the general procedure, the reaction of *N,N*-diethyl-4-hexylbenzamide (0.30 mmol), EtOD-*d_1_* (9.0 mmol) and Na dispersion in oil (3.0 mmol), after chromatography (0–25% EtOAc/petroleum ether), afforded **4a**, 44.9 mg, 77% yield as a colorless oil. Spectroscopic properties matched those described previously [79].

**(4-Hexylphenyl)methan-*d_2_*-ol (Table 5, 4a).** According to the general procedure, the reaction of 4-hexyl-*N,N*-diisopropylbenzamide (0.30 mmol), EtOD-*d_1_* (9.0 mmol) and Na dispersion in oil (3.0 mmol), after chromatography (0–25% EtOAc/petroleum ether), afforded **4a**, 34.4 mg, 59% yield as a colorless oil. Spectroscopic properties matched those described previously [79].

**(4-Hexylphenyl)methan-*d_2_*-ol (Table 5, 4a).** According to the general procedure, the reaction of 4-hexyl-*N*-methyl-*N*-phenylbenzamide (0.30 mmol), EtOD-*d_1_* (9.0 mmol) and Na dispersion in oil (3.0 mmol), after chromatography (0–25% EtOAc/petroleum ether), afforded **4a**, 52.5 mg, 90% yield as a colorless oil. Spectroscopic properties matched those described previously [79].

**(4-Hexylphenyl)methan-*d_2_*-ol (Table 5, 4a).** According to the general procedure, the reaction of *N*-benzyl-4-hexyl-*N*-methylbenzamide (0.30 mmol), EtOD-*d_1_* (9.0 mmol) and Na dispersion in oil (3.0 mmol), after chromatography (0–25% EtOAc/petroleum ether), afforded **4a**, 39.1 mg, 67% yield as a colorless oil. Spectroscopic properties matched those described previously [79].

**(4-Cyclohexylphenyl)methan-*d_2_*-ol (**Table 5**, 4b)**. According to the general procedure, the reaction of (4-cyclohexylphenyl)(morpholino)methanone (0.30 mmol), EtOD-*d_1_* (9.0 mmol) and Na dispersion in oil (3.0 mmol), after chromatography (0–20% EtOAc/petroleum ether), afforded 4b, 46.1 mg, 80% yield as a white solid. ^1^H NMR (300 MHz, CDCl_3_) δ 7.28 (m, 2H), 7.20 (m, 2H), 2.50 (m, 1H), 1.93–1.64 (m, 7H), 1.48–1.33 (m, 4H). ^13^C NMR (75 MHz, CDCl_3_) δ 147.8, 138.3, 127.3, 127.1, 64.8 (m), 44.4, 34.6, 27.0, 26.3. Spectroscopic properties matched those described previously [80].

**(4-Isopropylphenyl)methan-*d_2_*-ol (**Table 5**, 4c).** According to the general procedure, the reaction of (4-isopropylphenyl)(morpholino)methanone (0.30 mmol), EtOD-*d_1_* (9.0 mmol) and Na dispersion in oil (3.0 mmol), after chromatography (0-25% EtOAc/petroleum ether), afforded 4c, 32.0 mg, 70% yield as a white solid. ^1^H NMR (300 MHz, CDCl_3_) δ 7.29 (m, 2H), 7.22 (m, 2H), 2.91 (m, 1H), 1.70 (br, 1H), 1.25 (d, *J* = 7.0 Hz, 6H). ^13^C NMR (75 MHz, CDCl_3_) δ 148.6, 138.3, 127.3, 126.7, 65.0 (m), 34.0, 24.1. Spectroscopic properties matched those described previously [81].

**(3-Hexylphenyl)methan- *d_2_*-ol (**Table 5**, 4d).** According to the general procedure, the reaction of (3-hexylphenyl)(morpholino)methanone (0.30 mmol), EtOD-*d_1_* (9.0 mmol) and Na dispersion in oil (3.0 mmol), after chromatography (0-25% EtOAc/petroleum ether), afforded 4d, 29.3 mg, 50% yield as a colorless oil. *New compound*. ^1^H NMR (400 MHz, CDCl_3_) δ 7.30–7.25 (m, 1H), 7.21–7.15 (m, 2H), 7.14–7.10 (m, 2H), 2.61 (t, *J* = 7.7 Hz, 2H), 1.67–1.56 (m, 3H), 1.35–1.25 (m, 6H), 0.88 (t, *J* = 6.8 Hz, 3H). ^13^C NMR (100 MHz, CDCl_3_) δ 143.4, 140.7, 128.5, 127.9, 127.2, 124.4, 77.4, 77.1, 76.7, 36.0, 31.8, 31.5, 29.1, 22.6, 14.2. HRMS (ESI/Q-TOF) m/z: [M + Na]^+^ calcd for C_13_H_18_D_2_ONa 217.1537 found 217.1526.

**General procedure for one-pot sequential process**. An oven-dried vial equipped with a stir bar was charged with an amide substrate (neat, 0.25 mmol, 1.0 equiv) and Fe(acac)_3_ (0.1 mol%), placed under a positive pressure of argon and subjected to three evacuation/backfilling cycles under vacuum. 2-Methyltetrahydrofuran (1.0 M) and DMI (neat, 200 mol%) were sequentially added with vigorous stirring at room temperature, the reaction mixture was cooled to 0 °C, a solution of Grignard reagent (typically, 1.05 equiv) was added dropwise with vigorous stirring and the reaction mixture was stirred for 18 h at 0 °C. After the indicated time, the reaction mixture was diluted with HCl (1.0 *N*, 0.5 mL) and EtOAc (1 × 15 mL), the organic layer was extracted with HCl (1.0 *N*, 2 × 5 mL), dried and concentrated. The resulting residue was then dissolved in hexane (2 mL), EtOH (7.5 mmol) was added, followed by sodium dispersions in oil (34 wt%, 2.5 mmol) under Ar at 0 °C and the resulting solution was stirred vigorously. After 20 min, the reaction mixture was quenched by an aqueous solution of NaHCO_3_ (4.0 mL, saturated) and the reaction mixture was diluted with EtOAc (10 mL) and brine (10 mL). The aqueous layer was extracted with EtOAc (2 × 10 mL), the organic layers were combined, dried over MgSO_4_, filtered and concentrated. The crude product was purified by flash chromatography on silica gel (EtOAc/petroleum ether).

## Figures and Tables

**Table 1:**
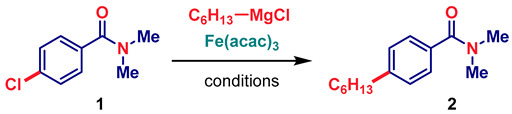
Optimization of Iron-Catalyzed Cross-Coupling ^1^.

Entry	Fe(acac)_3_ (mol%)	Ligand	Ligand (mol %)	Time (h)	Yield ^2^ (%)
1	-	-	-	18	<2
2	0.10	-	-	18	48
3	0.10	DMI	200	18	90
4 ^3^	0.10	DMI	200	18	88
5 ^4^	0.10	DMI	200	18	76
6 ^5^	0.10	DMI	200	18	95

^1^ Conditions: **1** (0.50 mmol), Fe(acac)_3_ (0.10 mol%), 2-MeTHF (1.0 M), C_6_H_13_MgCl (1.20 equiv, 2.0 M, THF), 0 °C, 18 h. C_6_H_13_MgCl added dropwise over 1–2 s. ^2^ Yield determined by ^1^H NMR and/or GC-MS. ^3^ THF instead of 2-MeTHF. ^4^ C_6_H_13_MgCl added at −78 °C. ^5^ C_6_H_13_MgCl (1.05 equiv).

**Table 2:**
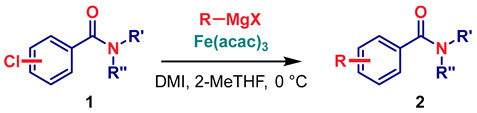
Iron-Catalyzed C(sp^2^)–C(sp^3^) Cross-Coupling of Chlorobenzamides with Alkyl Grignards ^1^.

Entry	2	Product	Yield (%)
1	**2a**	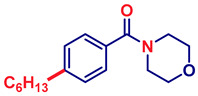	92
2	**2b**	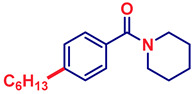	92
3 ^2^	**2c**	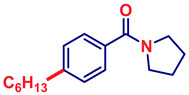	88
4	**2d**	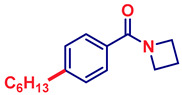	73
5	**2e**	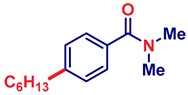	95
6 ^2^	**2f**	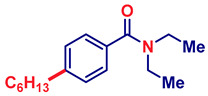	98
7 ^2^	**2g**	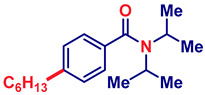	98
8	**2h**	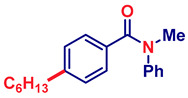	85
9	**2i**	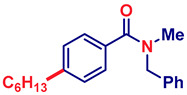	95
10 ^3^	**2j**	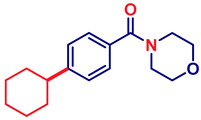	80
11 ^3^	**2k**	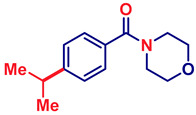	56
12	**2l**	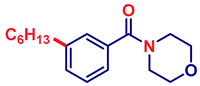	68

^1^ Conditions: **1** (0.50 mmol), Fe(acac)_3_ (0.10 mol%), 2-MeTHF (1.0 M), DMI (200 mol%), RMgX (1.05 equiv, THF), 0 °C, 18 h. ^2^ RMgX (1.20 equiv). ^3^ RMgX (2.0 equiv). See the Experimental Section for details.

**Table 3:**
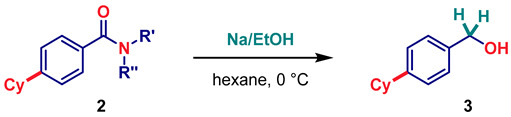
Optimization of Sodium Dispersion Mediated Reduction of Aromatic Amides ^1^.

Entry	Na (equiv)	EtOH (equiv)	Time (min)	Yield ^2^ (%)
1	10	30	20	81
2	10	20	20	72
3	10	40	20	75
4	10	50	20	65
5	5	15	20	68

^1^ Conditions: sodium dispersion (34.5 wt%, particle size 5–10 μm, 10 equiv), **2** (0.30 mmol), EtOH (15–50 equiv), hexane, 0 °C, 20 min. Na dispersion added dropwise to a solution of **2** and EtOH. ^2^ Yield determined by ^1^H NMR.

**Table 4:** Chemoselective Sodium Dispersion Mediated Reduction of Aromatic Amides to Benzyl Alcohols ^1^.

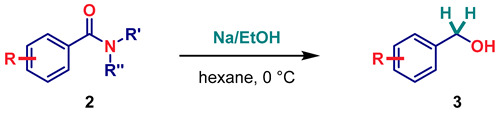		
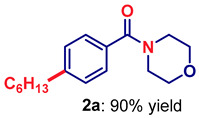	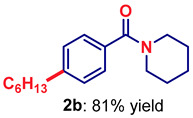	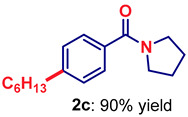
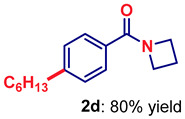	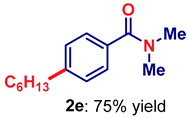	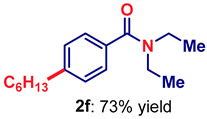
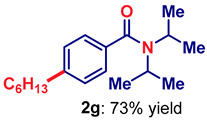	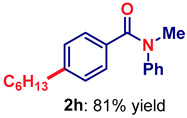	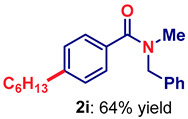
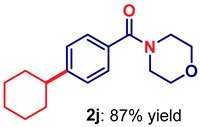	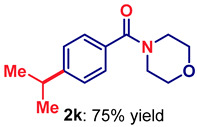	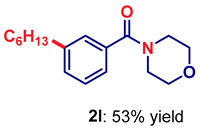

^1^ Conditions: sodium dispersion (34.5 wt%, particle size 5–10 μm, 10 equiv), **2** (0.30 mmol), EtOH (30 equiv), hexane, 0 °C, 20 min. Sodium dispersion added dropwise to a solution of **2** and EtOH.

**Table 5:** Chemoselective Sodium Dispersion Mediated Reductive Deuteration of Aromatic Amides to [D_2_]-Benzyl Alcohols ^1^.

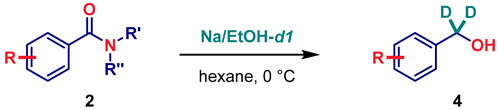		
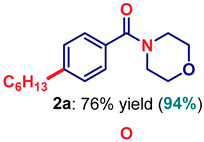	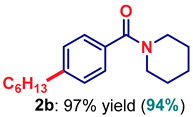	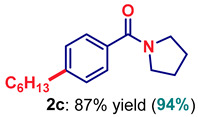
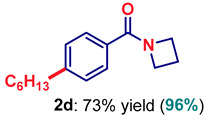	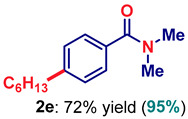	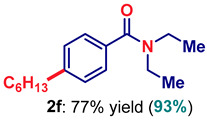
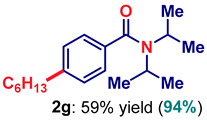	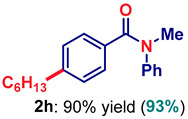	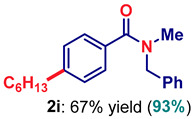
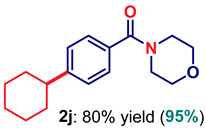	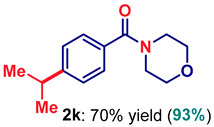	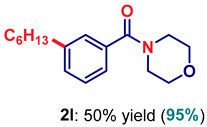

^1^ Conditions: sodium dispersion (34.5 wt%, particle size 5–10 μm, 10 equiv), **2** (0.30 mmol), EtOH-*d^1^* (30 equiv), hexane, 0 °C, 20 min. Na dispersion added dropwise to a solution of **2** and EtOH-*d^1^*. [D_2_]-incorporation is shown in brackets.

## Data Availability

Not applicable.

## Supplementary Materials

^1^H and ^13^C NMR spectra are available online at https://www.mdpi.com/article/10.3390/molecules28010223/s1.

## Sample Availability

Samples of all obtained compounds are available from the authors.
