# Supplementary material for: Sequential Iron-Catalyzed C(sp2)–C(sp3) Cross-Coupling of Chlorobenzamides/Chemoselective Amide Reduction and Reductive Deuteration to Benzylic Alcohols
Source: Molecules. 2022 Dec 27;28(1):223. doi: 10.3390/molecules28010223 (PMC9821805; doi:10.3390/molecules28010223)
Supplement: Supplementary file 1 [file molecules-28-00223-s001.zip › molecules-2106333-supplementary.pdf]

Sequential Iron-Catalyzed C(sp^2)–C(sp^3) Cross-Coupling of Chlorobenzamides/Chemoselective Amide Reduction and Reductive Deuteration to Benzylic Alcohols

Elwira Bisz[1,*], Pamela Podchorodecka[1], Hengzhao Li[2], Wioletta Ochędzan-Siodłak[1], Jie An[2,*] and Michal Szostak[3,*]

[1]*Department of Chemistry, Opole University, 48 Oleska Street, 45-052 Opole, Poland*
[2]*Department of Nutrition and Health, China Agricultural University, Beijing 100193, China*
[3]*Department of Chemistry, Rutgers University, 73 Warren Street, Newark, NJ 07102, USA*

ebisz@uni.opole.pl; jie_an@cau.edu.cn; michal.szostak@rutgers.edu

Supporting Information

Table of Contents S1
^{1}H and ^{13}C NMR Spectra S2

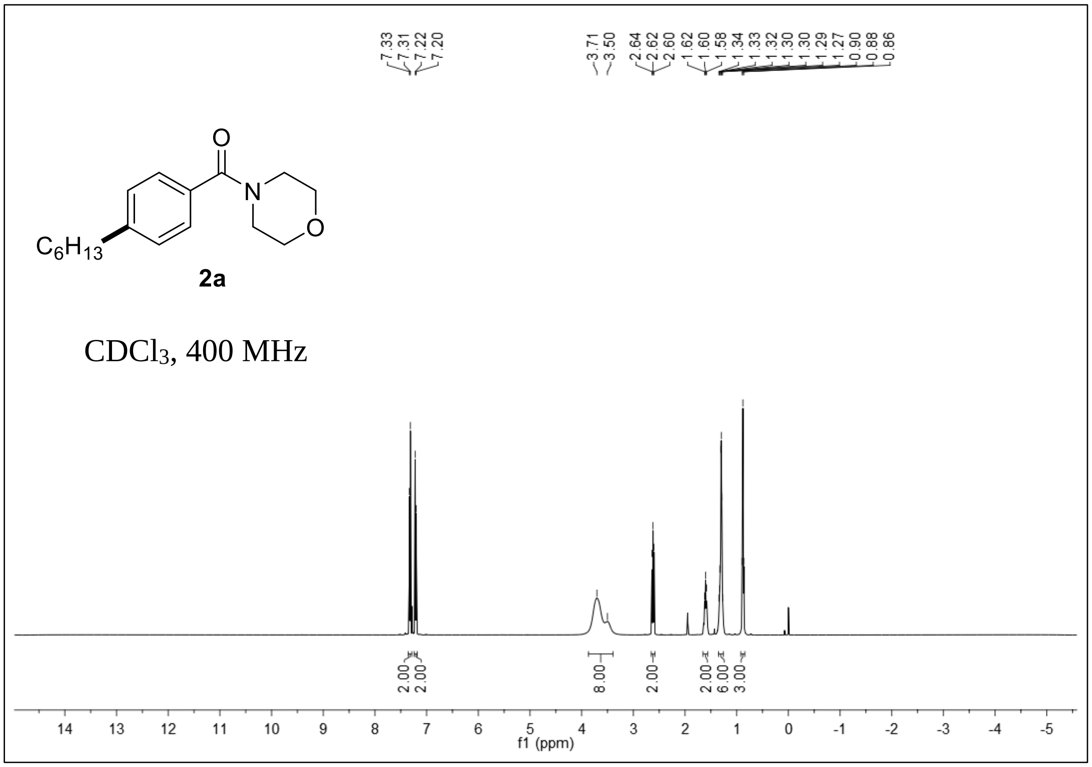

^{1}H NMR (400 MHz, $CDCl_3$) δ 7.32 (d, *J* = 8.2 Hz, 2H), 7.21 (d, *J* = 8.2 Hz, 2H), 3.93–3.36 (m, 8H), 2.62 (t, *J* = 7.7 Hz, 2H), 1.65–1.55 (m, 2H), 1.37–1.25 (m, 6H), 0.88 (t, *J* = 6.8 Hz, 3H).

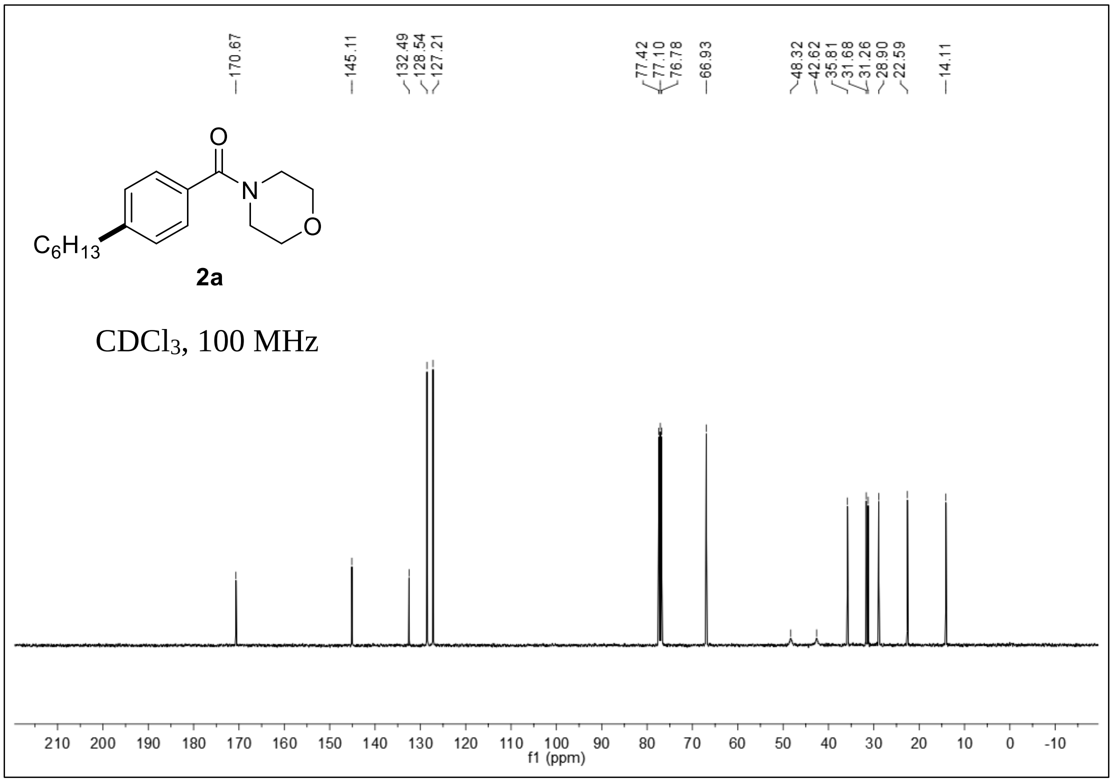

^{13}C NMR (100 MHz, $CDCl_3$) δ 170.67, 145.11, 132.49, 128.54, 127.21, 77.42, 77.10, 76.78, 66.93, 48.32, 42.62, 35.81, 31.68, 31.26, 28.90, 22.59, 14.11.

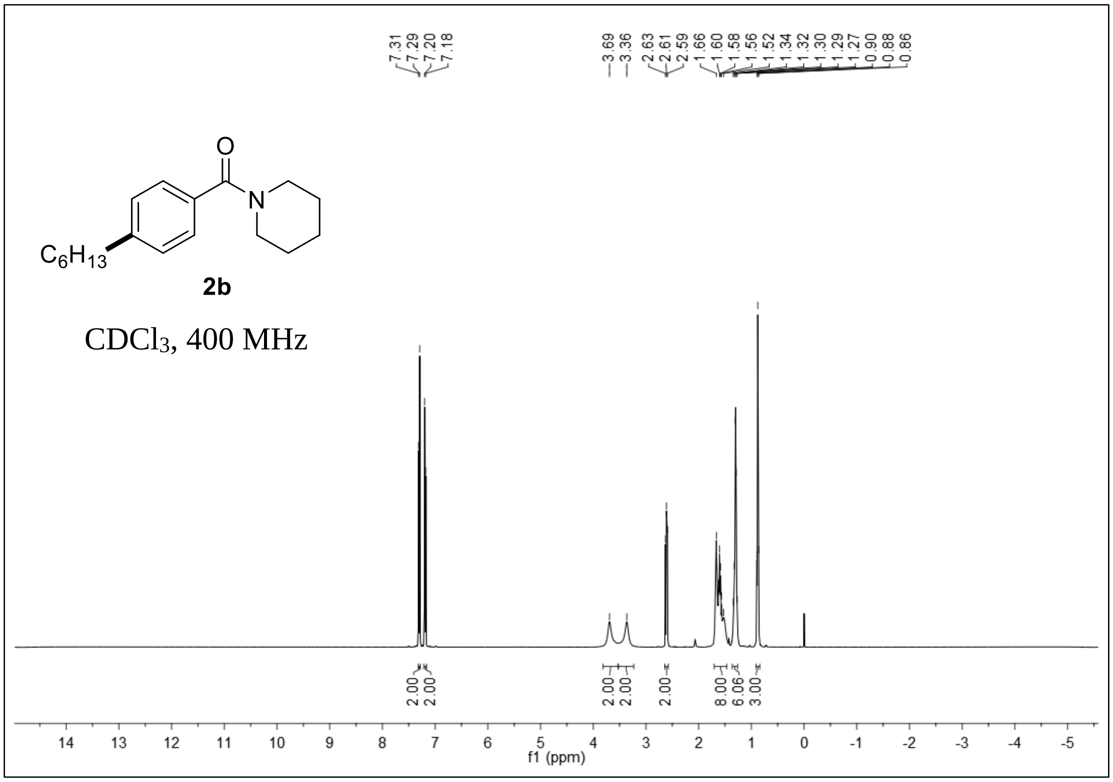

^{1}H NMR (400 MHz, $CDCl_3$) δ 7.30 (d, *J* = 8.2 Hz, 2H), 7.19 (d, *J* = 8.3 Hz, 2H), 3.69 (brs, 2H), 3.36 (brs, 2H), 2.61 (t, *J* = 7.7 Hz, 2H), 1.71–1.44 (m, 8H), 1.37–1.24 (m, 6H), 0.88 (t, *J* = 6.7 Hz, 3H).

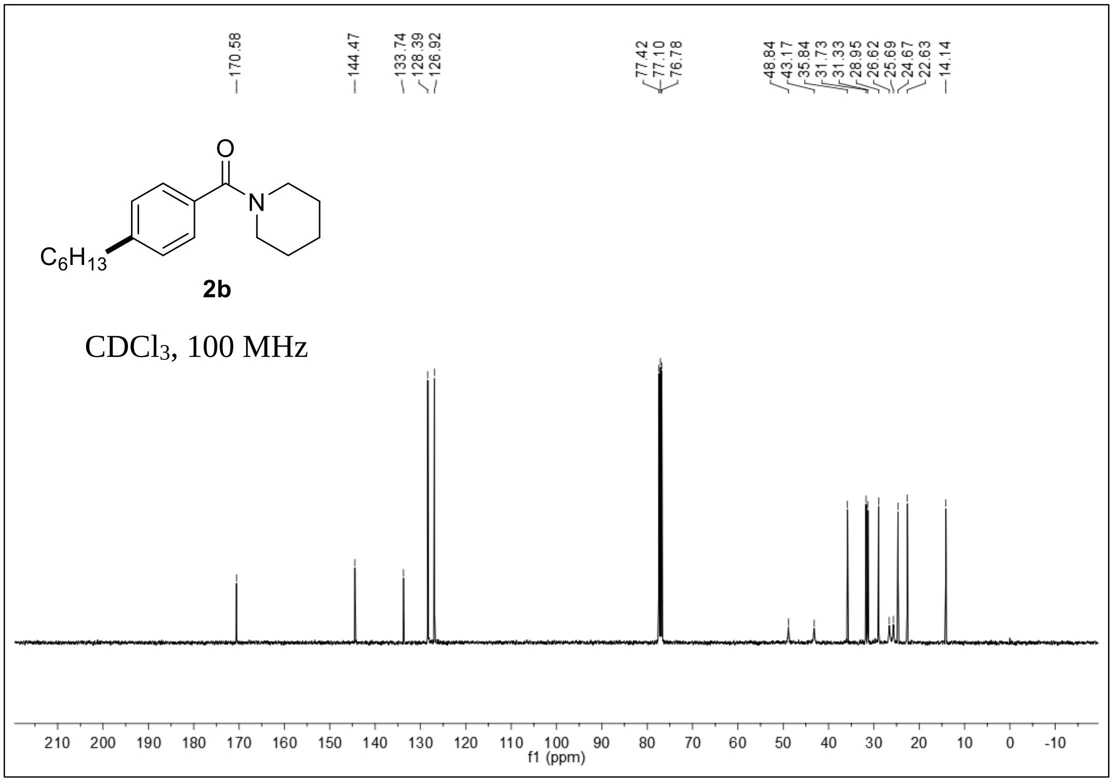

^{13}C NMR (100 MHz, $CDCl_3$) δ 170.58, 144.47, 133.74, 128.39, 126.92, 77.42, 77.10, 76.78, 48.84, 43.17, 35.84, 31.73, 31.33, 28.95, 26.62, 25.69, 24.67, 22.63, 14.14.

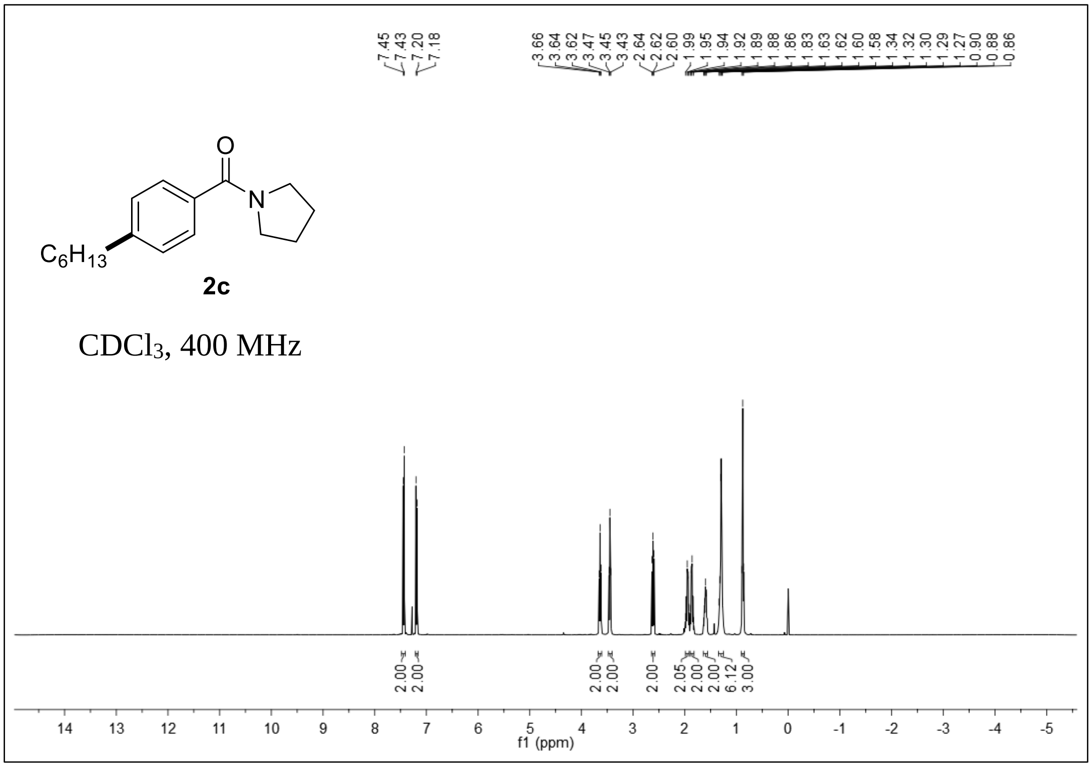

^{1}H NMR (400 MHz, $CDCl_3$) δ 7.44 (d, *J* = 8.2 Hz, 2H), 7.19 (d, *J* = 8.3 Hz, 2H), 3.64 (t, *J* = 7.0 Hz, 2H), 3.45 (t, *J* = 6.6 Hz, 2H), 2.62 (t, *J* = 7.7 Hz, 2H), 2.00–1.91 (m, 2H), 1.90–1.82 (m, 2H), 1.64–1.56 (m, 2H), 1.35–1.25 (m, 6H), 0.88 (t, *J* = 6.8 Hz, 3H).

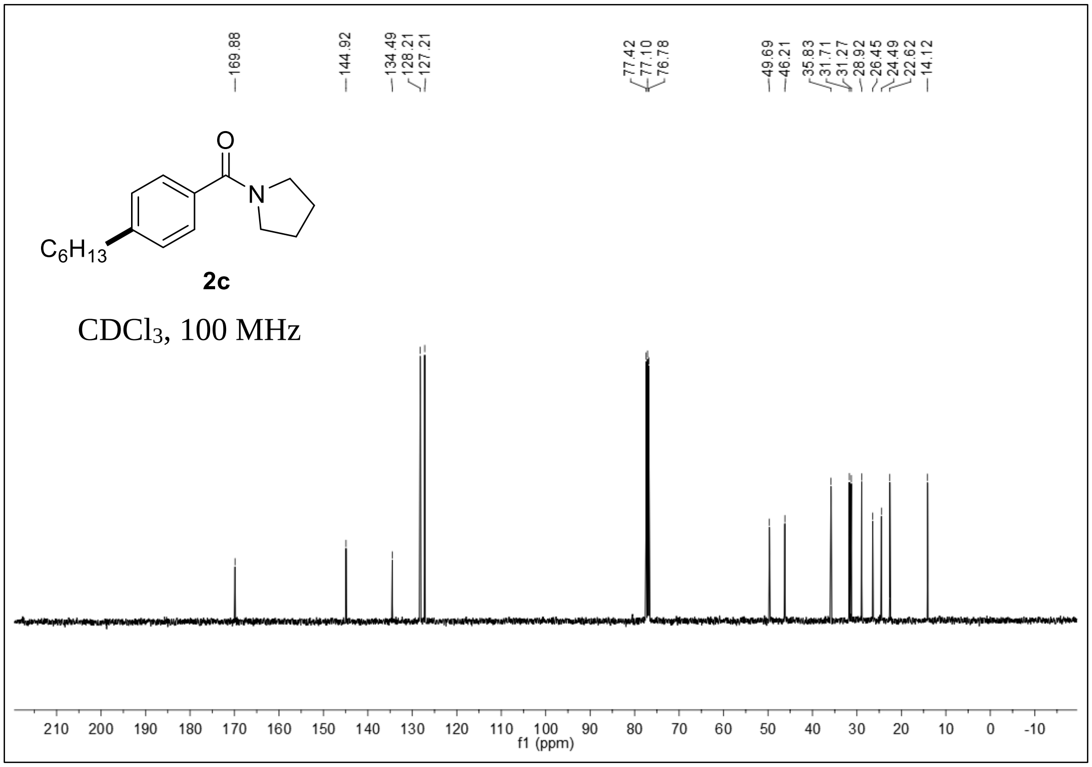

^{13}C NMR (100 MHz, $CDCl_3$) δ 169.88, 144.92, 134.49, 128.21, 127.21, 77.42, 77.10, 76.78, 49.69, 46.21, 35.83, 31.71, 31.27, 28.92, 26.45, 24.49, 22.62, 14.12.

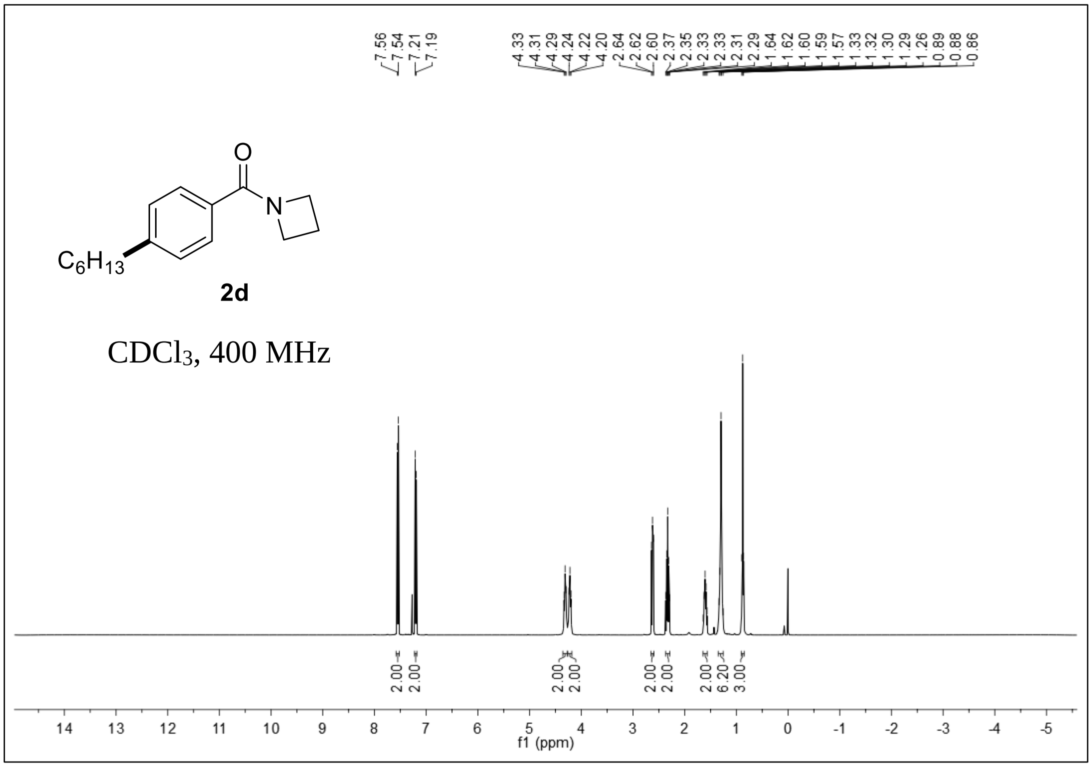

^{1}H NMR (400 MHz, $CDCl_3$) δ 7.55 (d, *J* = 8.3 Hz, 2H), 7.20 (d, *J* = 8.4 Hz, 2H), 4.31 (t, *J* = 7.5 Hz, 2H), 4.22 (t, *J* = 7.7 Hz, 2H), 2.62 (t, *J* = 7.7 Hz, 2H), 2.37–2.29 (m, 2H), 1.64–1.56 (m, 2H), 1.35–1.25 (m, 6H), 0.88 (t, *J* = 6.8 Hz, 3H).

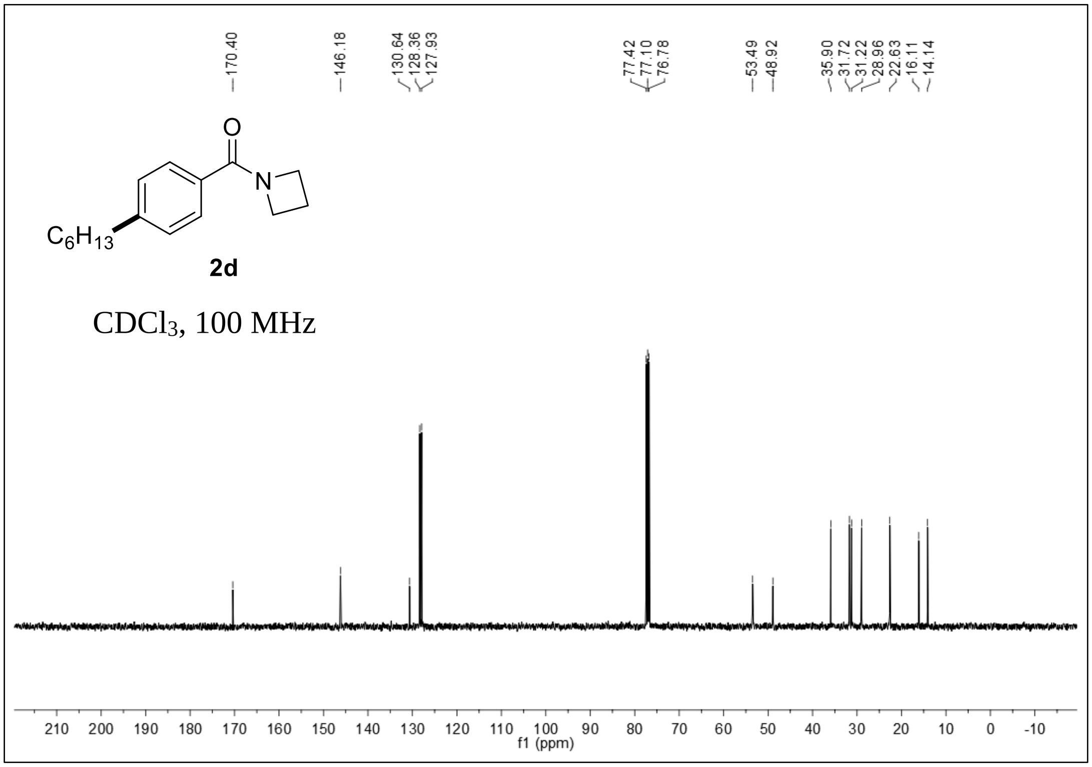

^{13}C NMR (100 MHz, $CDCl_3$) δ 170.40, 146.18, 130.64, 128.36, 127.93, 77.42, 77.10, 76.78, 53.49, 48.92, 35.90, 31.72, 31.22, 28.96, 22.63, 16.11, 14.14.

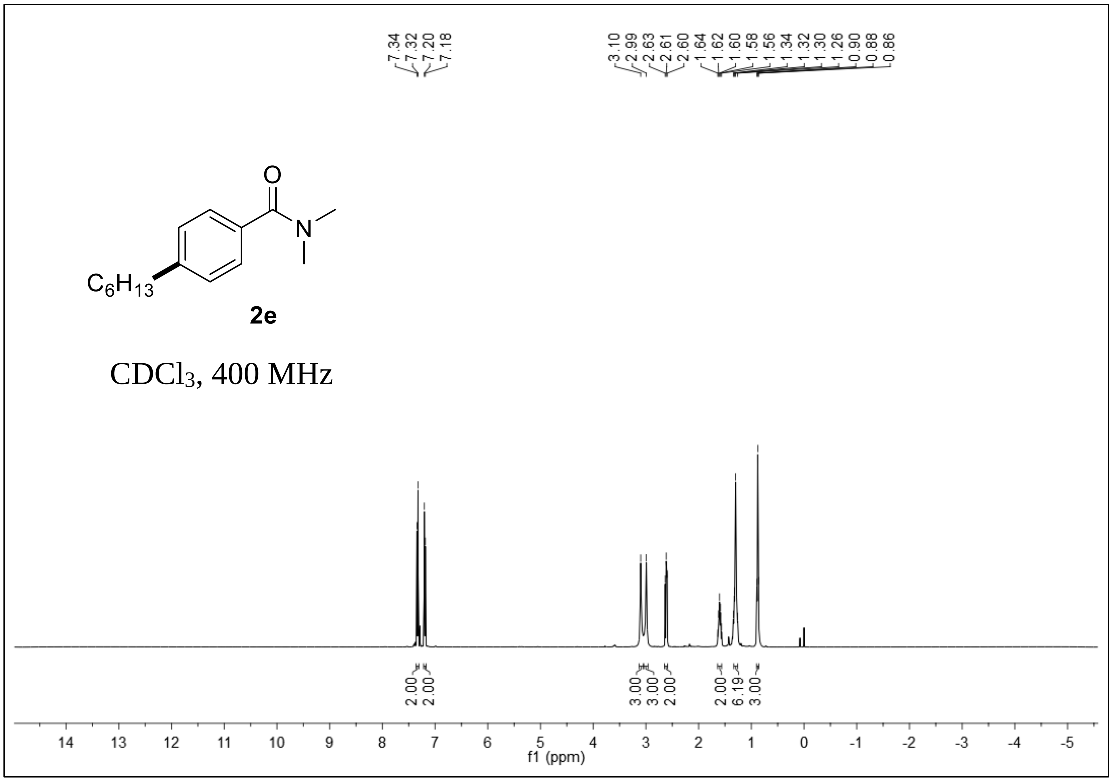

^{1}H NMR (400 MHz, $CDCl_3$) δ 7.33 (d, *J* = 8.1 Hz, 2H), 7.19 (d, *J* = 8.1 Hz, 2H), 3.10 (brs, 3H), 2.99 (brs, 3H), 2.61 (t, *J* = 7.7 Hz, 2H), 1.65–1.55 (m, 2H), 1.37–1.25 (m, 6H), 0.88 (t, *J* = 6.8 Hz, 3H).

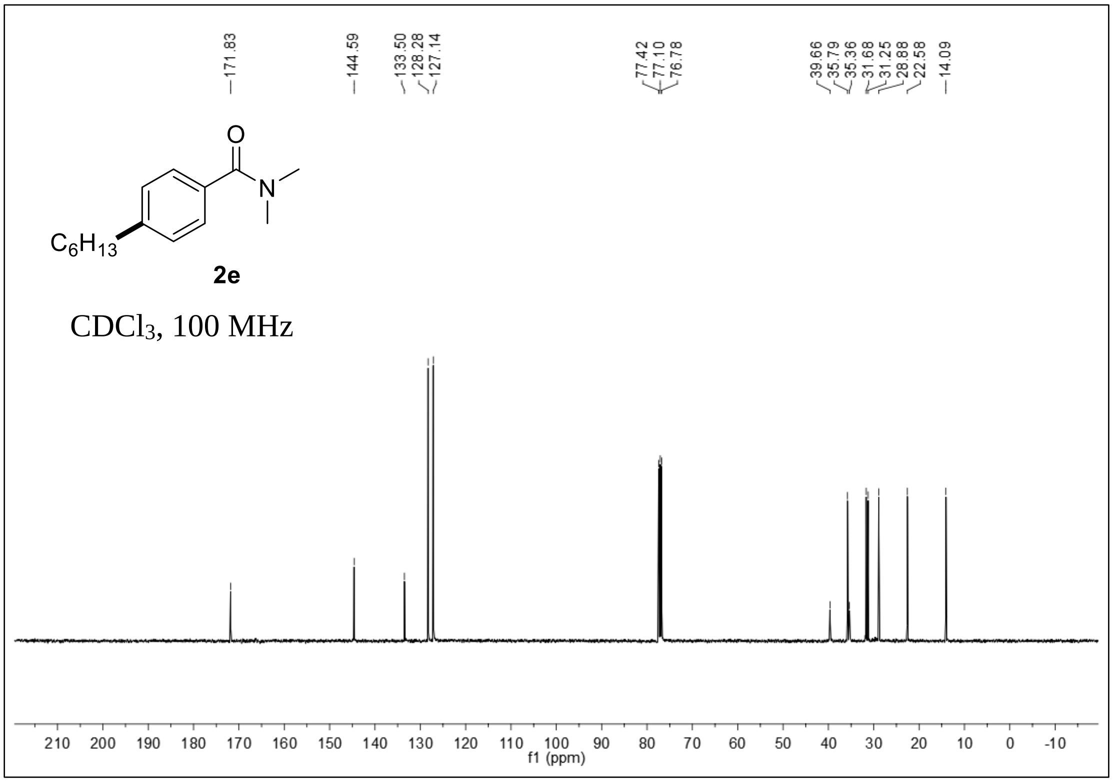

^{13}C NMR (100 MHz, $CDCl_3$) δ 171.83, 144.59, 133.50, 128.28, 127.14, 77.42, 77.10, 76.78, 39.66, 35.79, 35.36, 31.68, 31.25, 28.88, 22.58, 14.09.

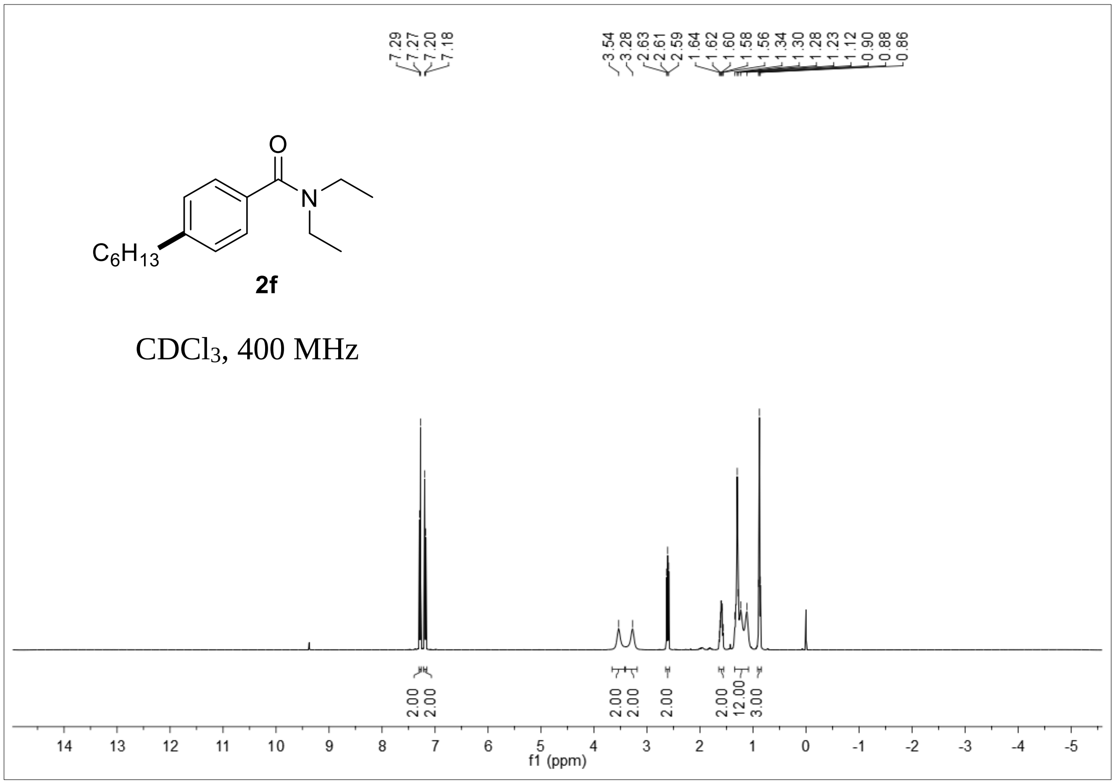

^{1}H NMR (400 MHz, $CDCl_3$) δ 7.28 (d, *J* = 8.1 Hz, 2H), 7.19 (d, *J* = 8.2 Hz, 2H), 3.54 (brs, 2H), 3.28 (brs, 2H), 2.61 (t, *J* = 7.7 Hz, 2H), 1.65–1.55 (m, 2H), 1.36–1.06 (m, 12H), 0.88 (t, *J* = 6.8 Hz, 3H).

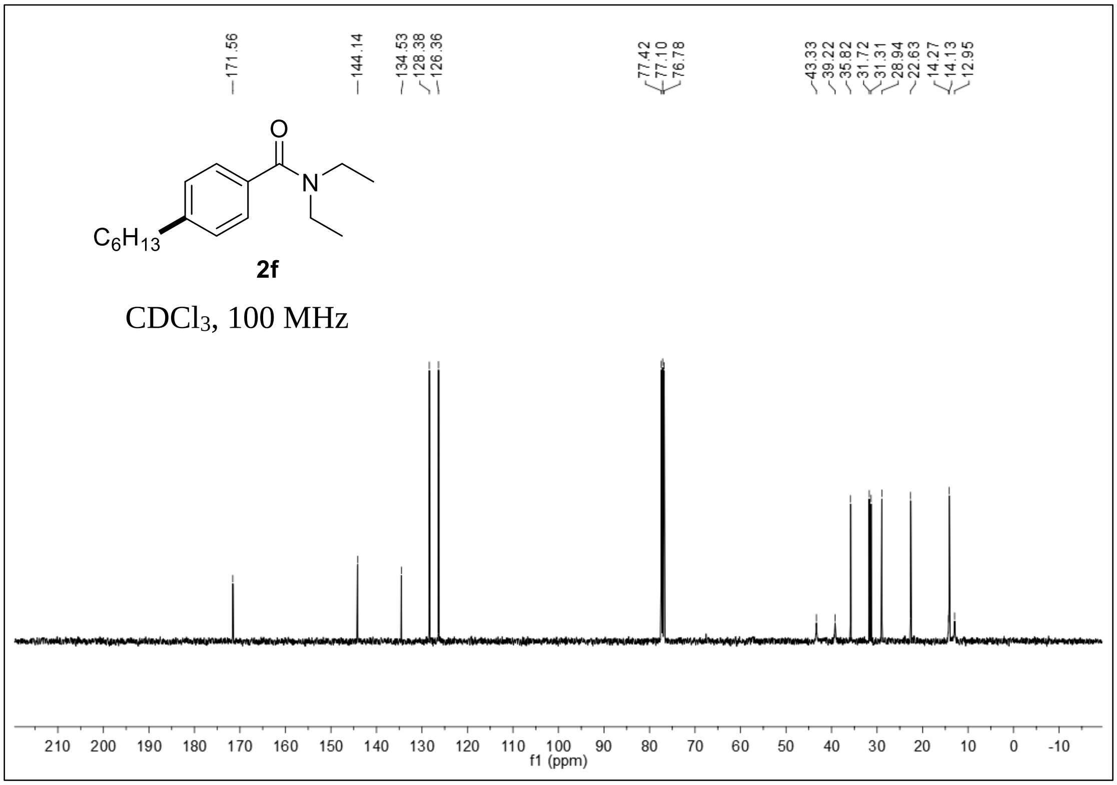

^{13}C NMR (100 MHz, $CDCl_3$) δ 171.56, 144.14, 134.53, 128.38, 126.36, 77.42, 77.10, 76.78, 43.33, 39.22, 35.82, 31.72, 31.31, 28.94, 22.63, 14.27, 14.13, 12.95.

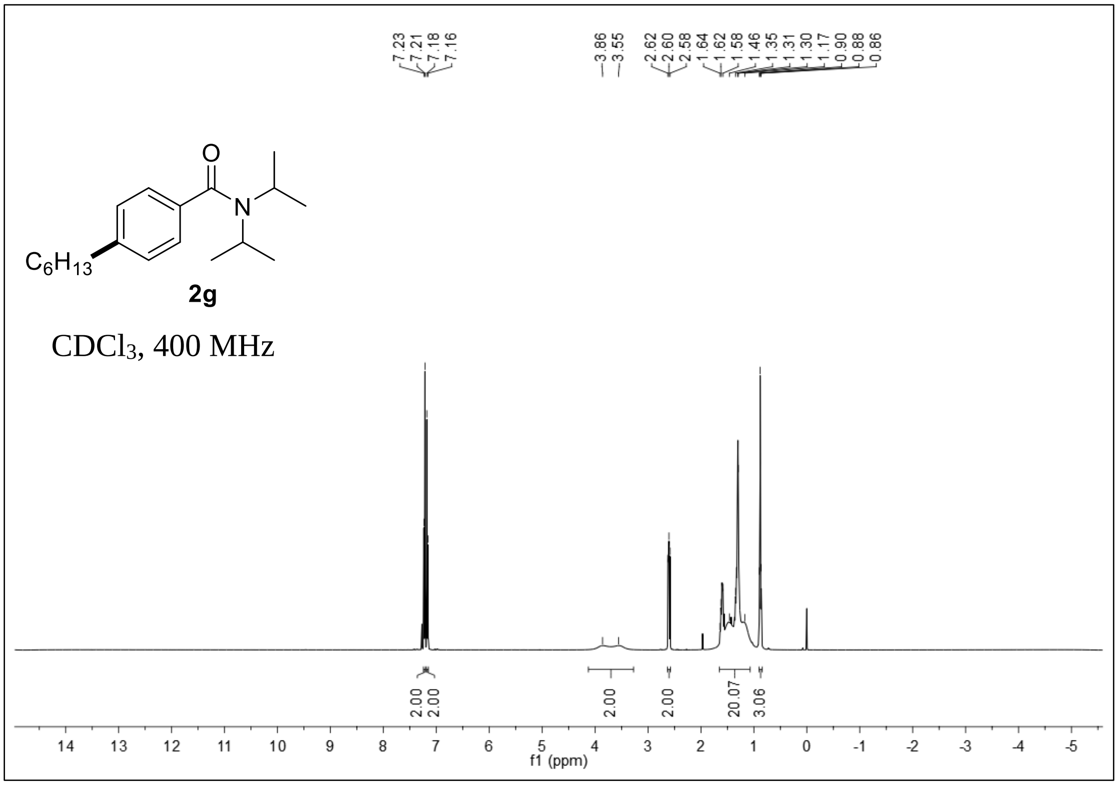

^{1}H NMR (400 MHz, $CDCl_3$) δ 7.22 (d, *J* = 8.2 Hz, 2H), 7.17 (d, *J* = 8.3 Hz, 2H), 4.11–3.27 (m, 2H), 2.60 (t, *J* = 7.7 Hz, 2H), 1.65–1.07 (m, 20 H), 0.88 (t, *J* = 6.8 Hz, 3H).

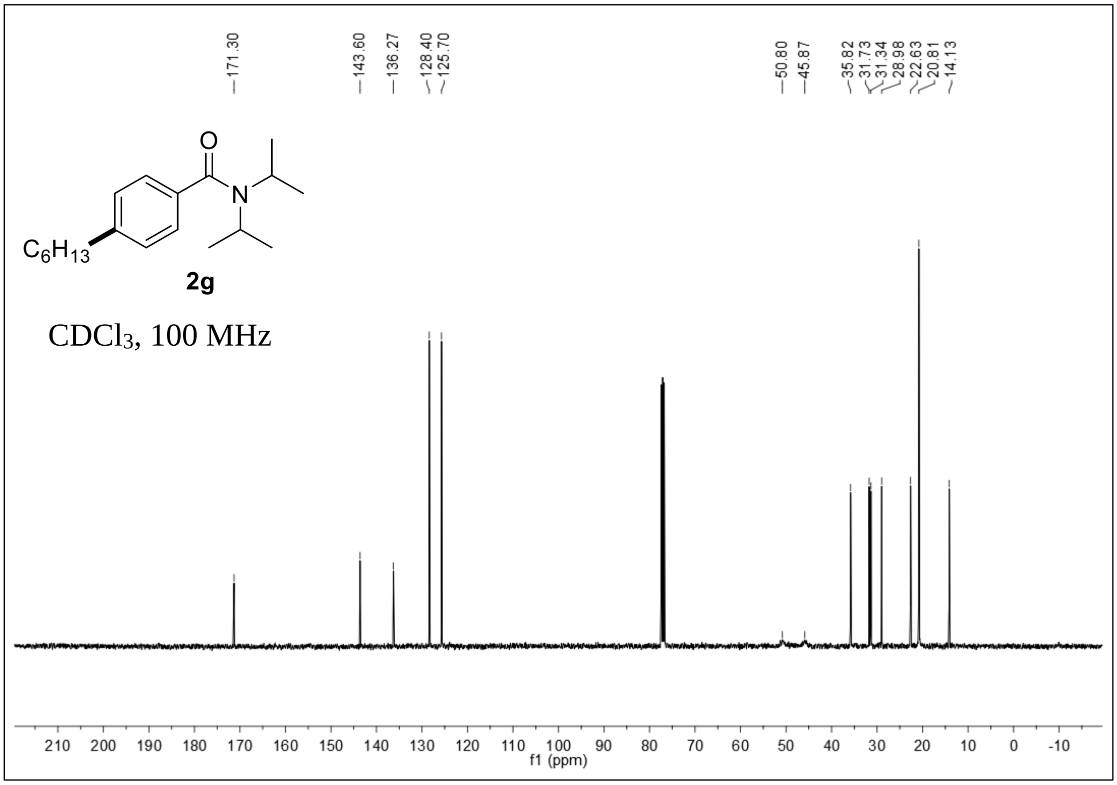

^{13}C NMR (100 MHz, $CDCl_3$) δ 171.30, 143.60, 136.27, 128.40, 125.70, 50.80, 45.87, 35.82, 31.73, 31.34, 28.98, 22.63, 20.81, 14.13.

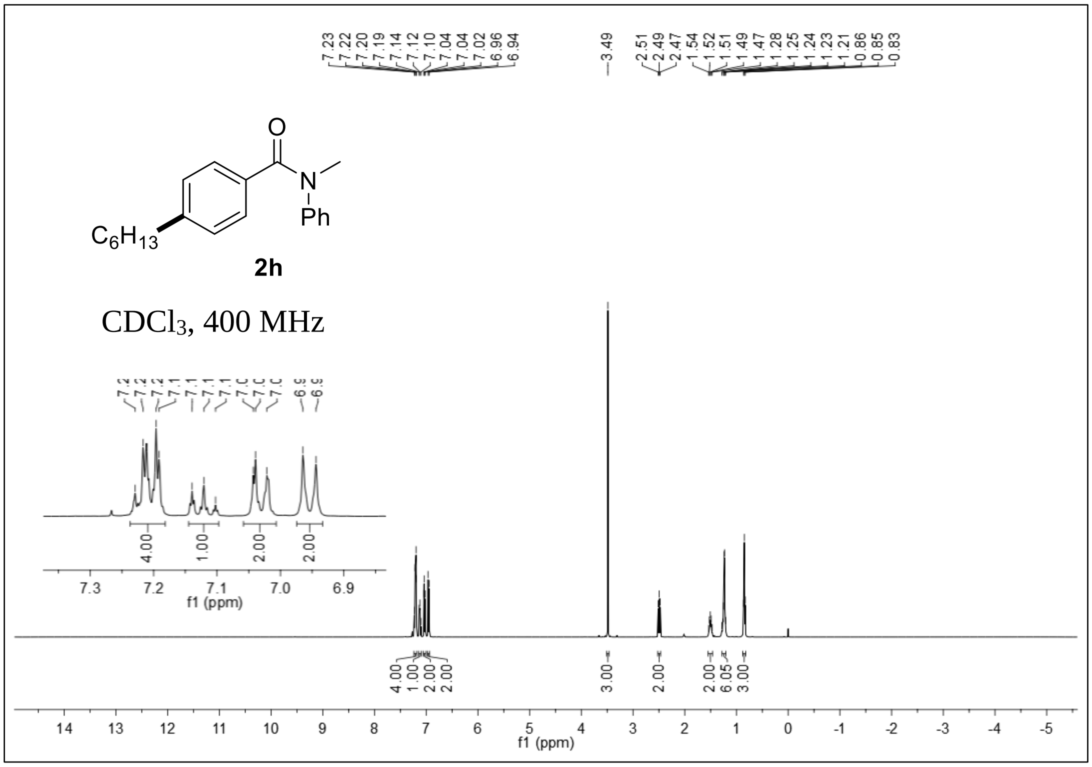

^{1}H NMR (400 MHz, CDCl3) δ 7.24–7.18 (m, 4H), 7.15–7.09 (m, 1H), 7.06–7.01 (m, 2H), 6.95 (d, *J* = 8.3 Hz, 2H), 3.49 (s, 3H), 2.49 (t, *J* = 7.7 Hz, 2H), 1.55–1.46 (m, 2H), 1.28–1.20 (m, 6H), 0.85 (t, *J* = 6.9 Hz, 3H).

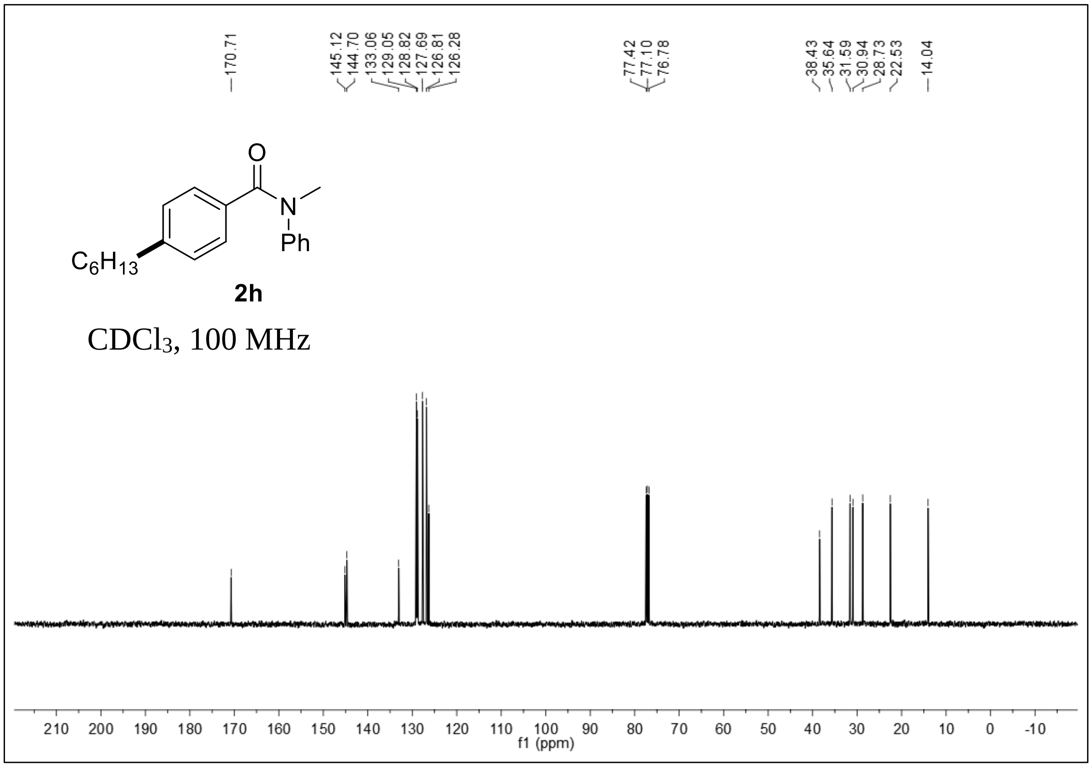

^{13}C NMR (100 MHz, $CDCl_3$) δ 170.71, 145.12, 144.70, 133.06, 129.05, 128.82, 127.69, 126.81, 126.28, 77.42, 77.10, 76.78, 38.43, 35.64, 31.59, 30.94, 28.73, 22.53, 14.04.

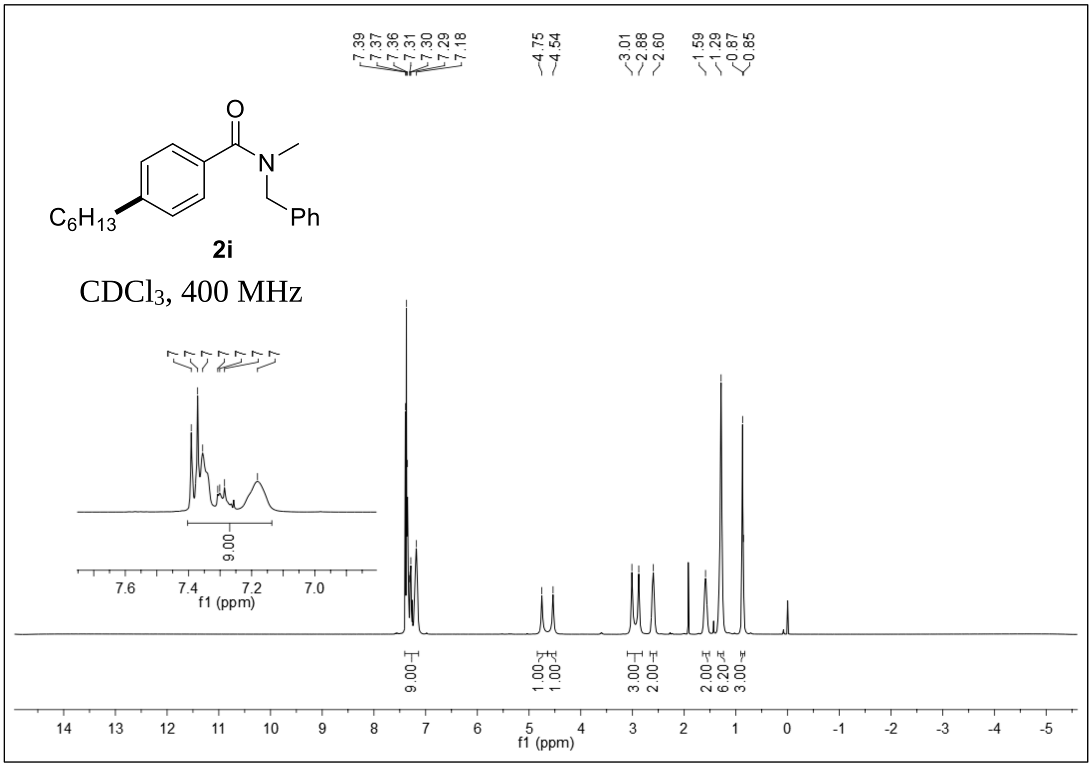

^{1}H NMR (400 MHz, CDCl3) δ 7.42–7.13 (m, 9H), 4.75 (brs, 1H), 4.54 (brs, 1H), 3.10–2.80 (m, 3H), 2.60 (brs, 2H), 1.59 (brs, 2H), 1.29 (brs, 6H), 0.87 (t, *J* = 6.8 Hz, 3H).

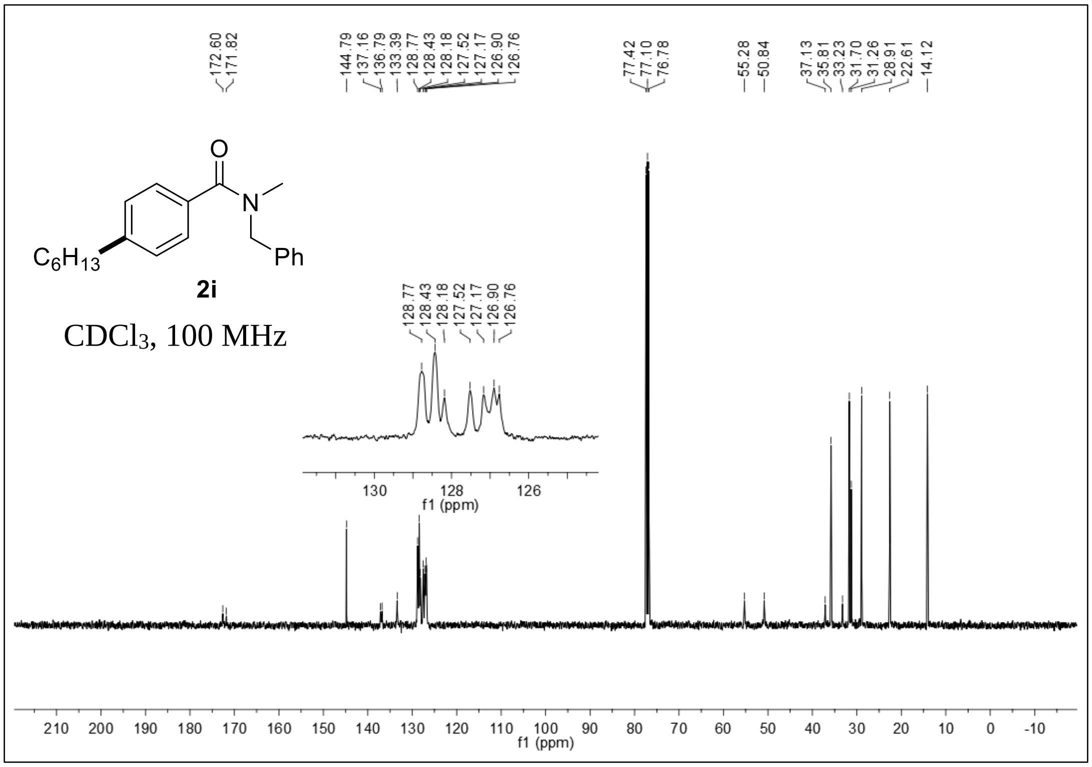

^{13}C NMR (100 MHz, $CDCl_3$) δ 172.60, 171.82, 144.79, 137.16, 136.79, 133.39, 128.77, 128.43, 128.18, 127.52, 127.17, 126.90, 126.76, 77.42, 77.10, 76.78, 55.28, 50.84, 37.13, 35.81, 33.23, 31.70, 31.26, 28.91, 22.61, 14.12.

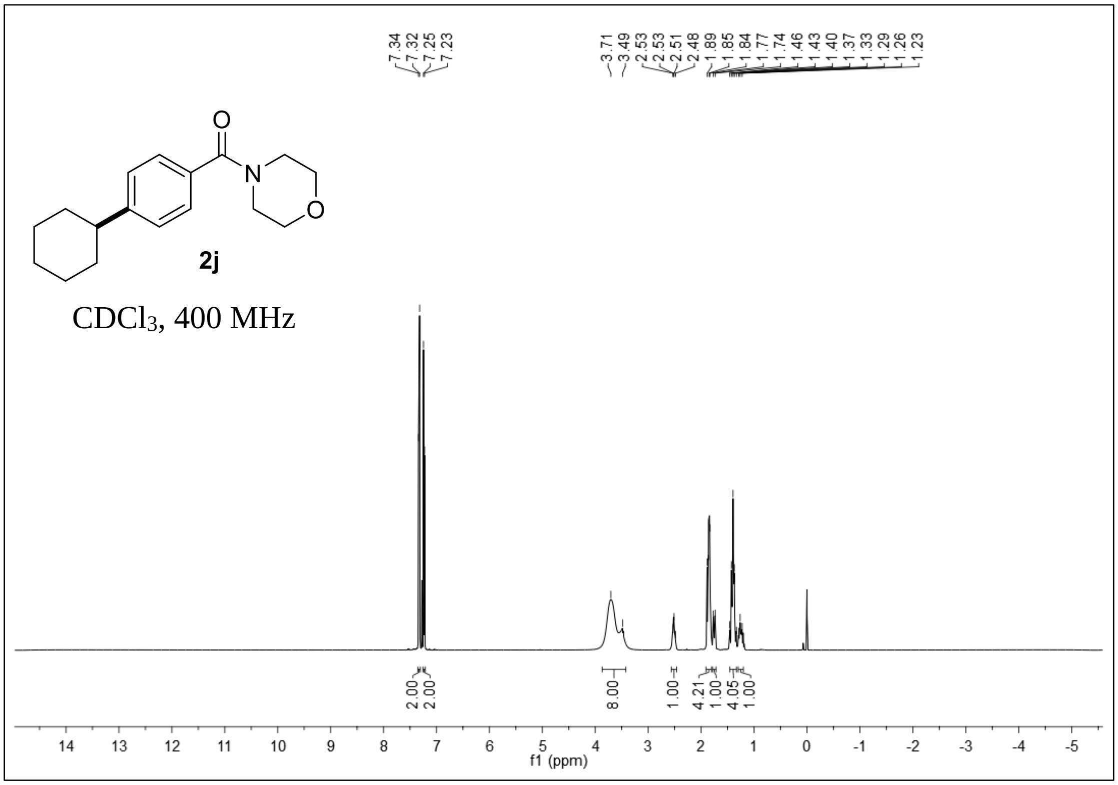

^{1}H NMR (400 MHz, CDCl3) δ 7.33 (d, *J* = 8.3 Hz, 2H), 7.24 (d, *J* = 8.0 Hz, 2H), 3.89–3.40 (m, 8H), 2.58–2.46 (m, 1H), 1.92–1.80 (m, 4H), 1.80–1.71 (m, 1H), 1.48–1.32 (m, 4H), 1.32–1.17 (m, 1H).

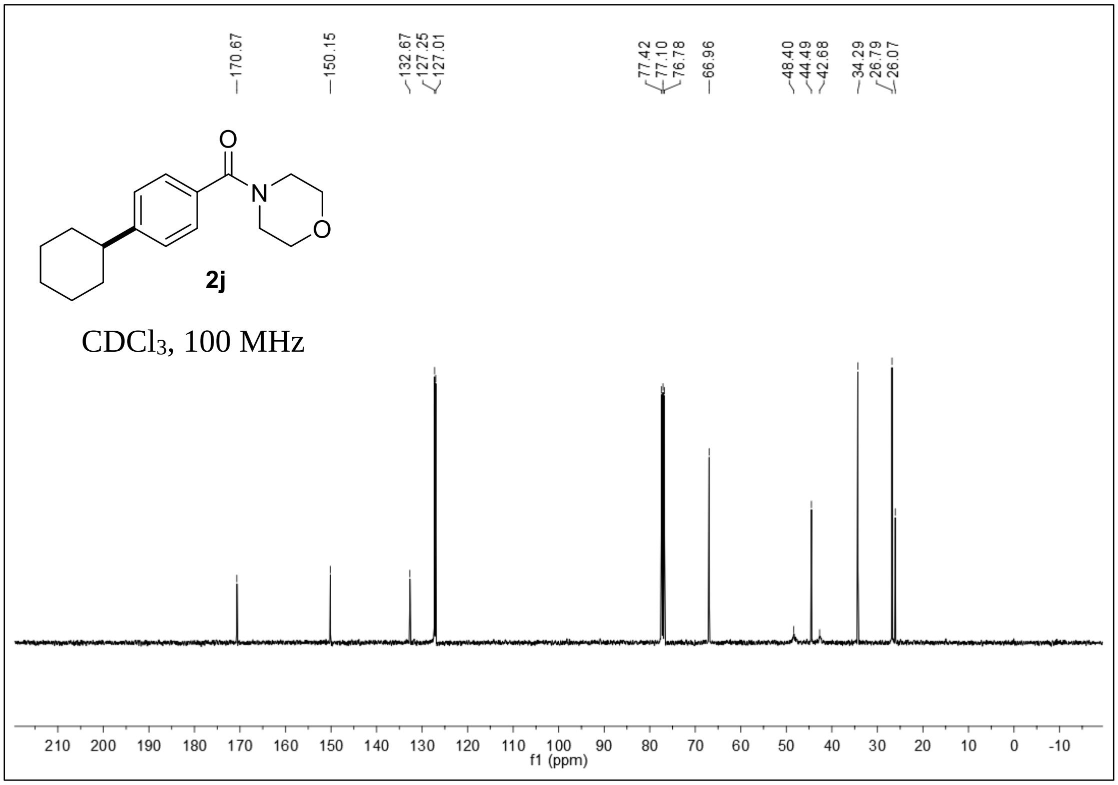

^{13}C NMR (100 MHz, $CDCl_3$) δ 170.67, 150.15, 132.67, 127.25, 127.01, 77.42, 77.10, 76.78, 66.96, 48.40, 44.49, 42.68, 34.29, 26.79, 26.07.

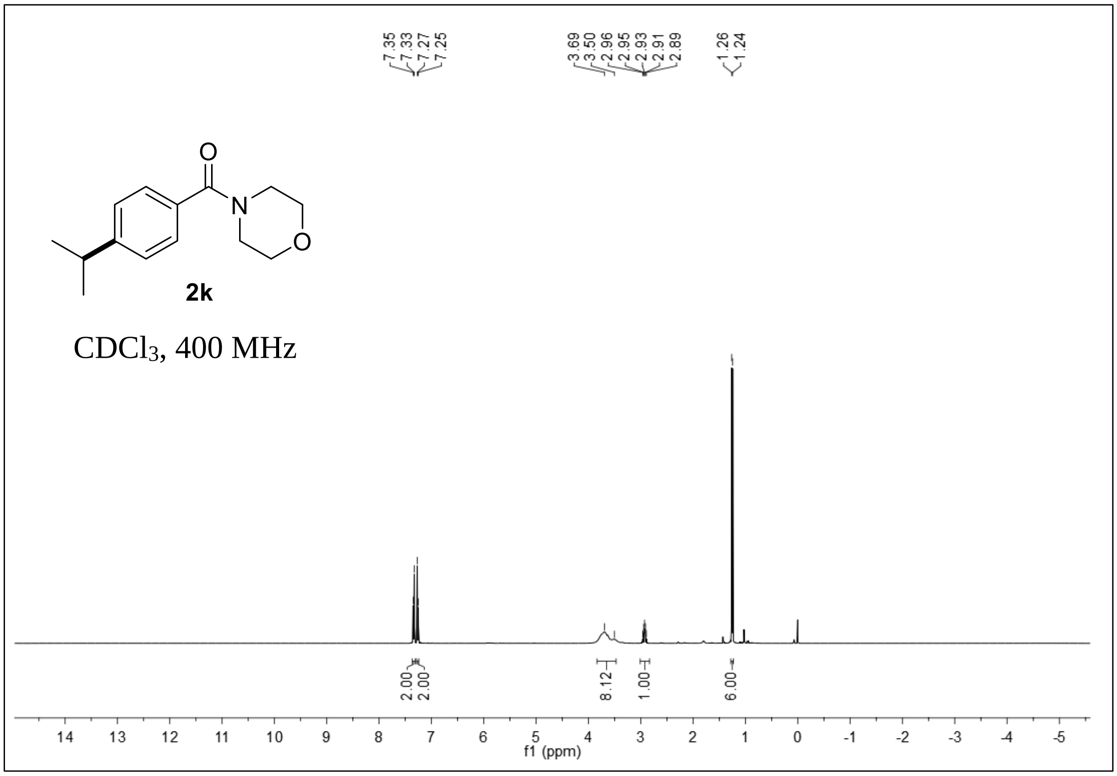

^{1}H NMR (400 MHz, $CDCl_3$) δ 7.34 (d, *J* = 8.3 Hz, 2H), 7.26 (d, *J* = 8.0 Hz, 2H), 3.85–3.44 (m, 8H), 2.99–2.85 (m, 1H), 1.25 (d, *J* = 6.9 Hz, 6H).

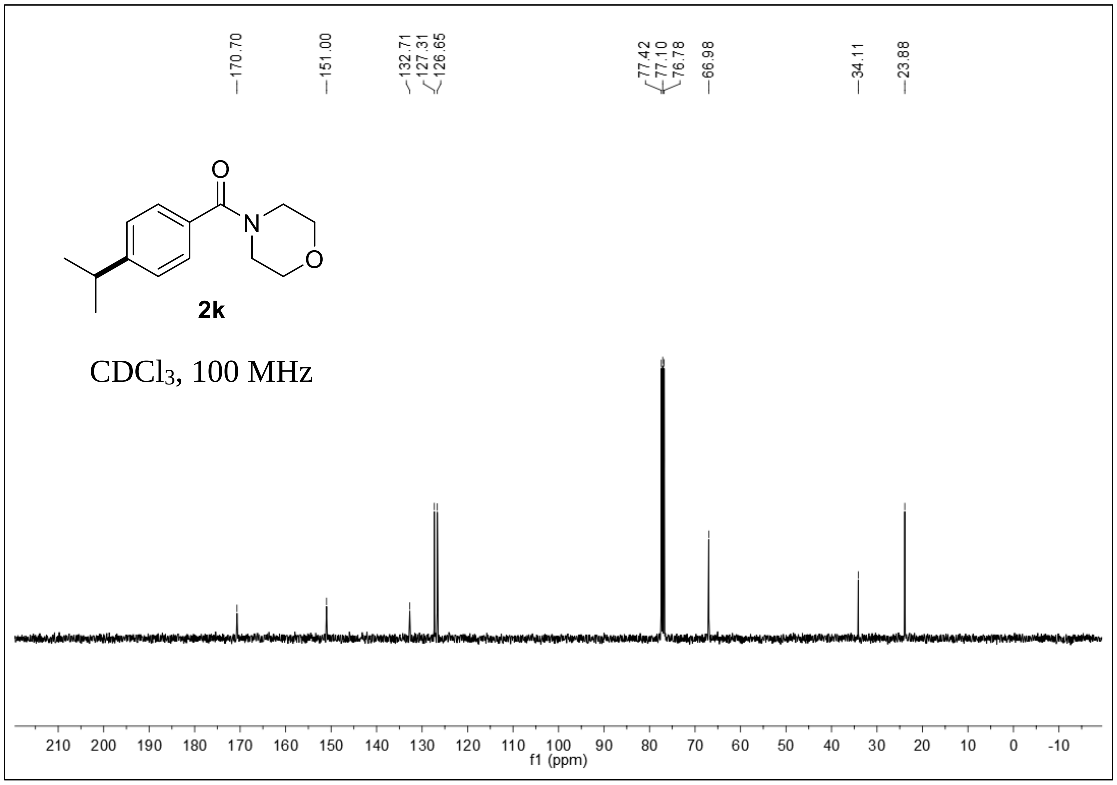

^{13}C NMR (100 MHz, $CDCl_3$) δ 170.70, 151.00, 132.71, 127.31, 126.65, 77.42, 77.10, 76.78, 66.98, 34.11, 23.88.

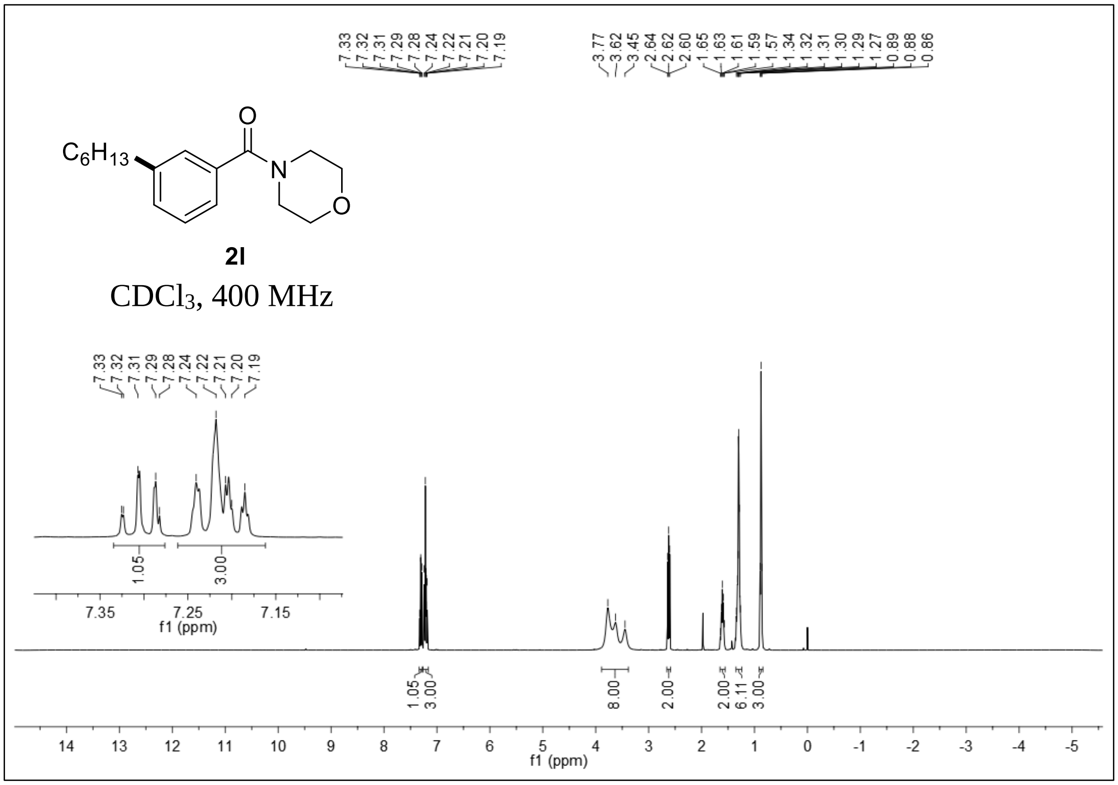

^{1}H NMR(400 MHz, CDCl3) δ 7.34–7.28 (m, 1H), 7.25–7.16 (m, 3H), 3.90–3.37 (m, 8H), 2.62 (t, *J* = 7.7 Hz, 2H), 1.65–1.55 (m, 2H), 1.35–1.24 (m, 6H), 0.88 (t, *J* = 6.9 Hz, 3H).

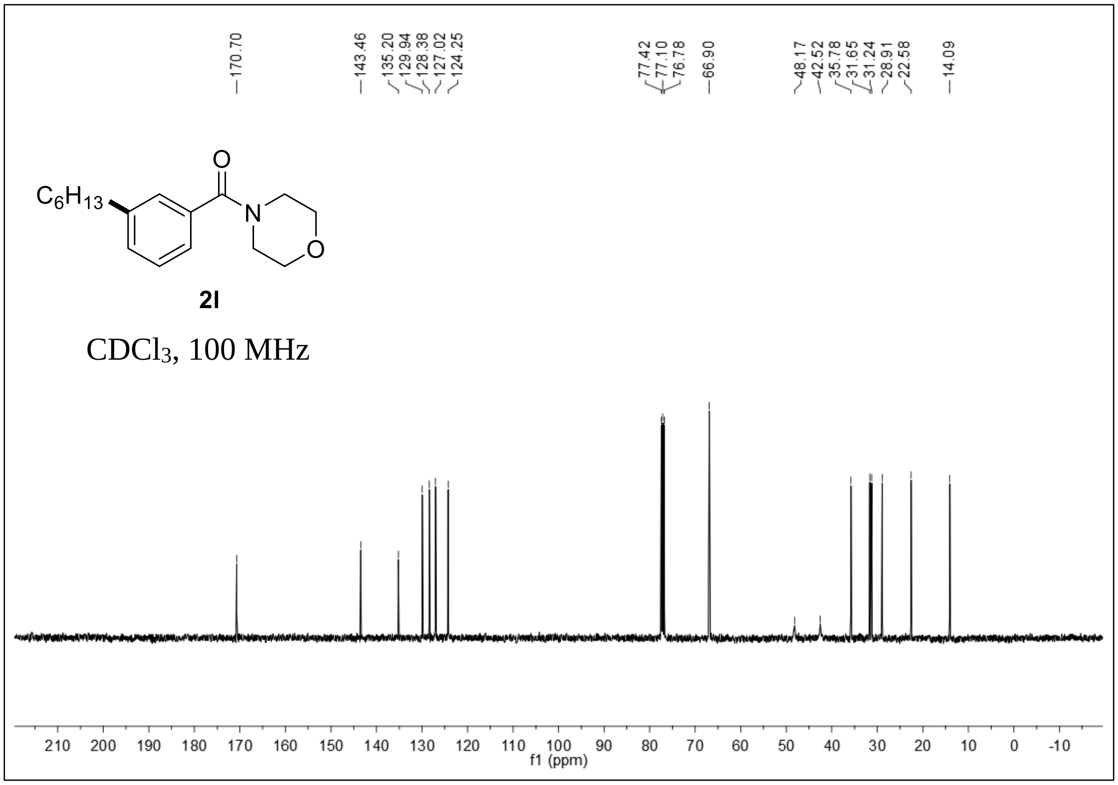

^{13}C NMR (101 MHz, $CDCl_3$) δ 170.70, 143.46, 135.20, 129.94, 128.38, 127.02, 124.25, 77.42, 77.10, 76.78, 66.90, 48.17, 42.52, 35.78, 31.65, 31.24, 28.91, 22.58, 14.09.

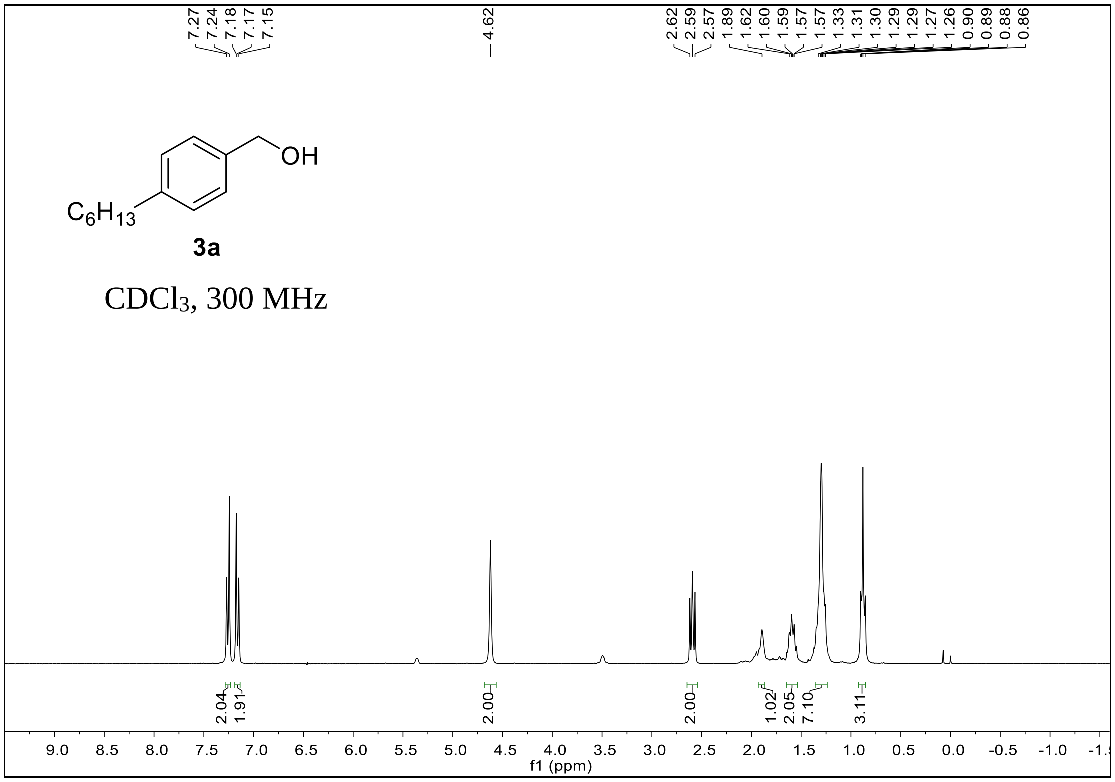

^{1}H NMR (300 MHz, $CDCl_3$) δ 7.27 (d, *J* = 7.9 Hz, 2H), 7.17 (d, *J* = 7.9 Hz, 2H), 4.62 (s, 2H), 2.59 (t, *J* = 7.7 Hz , 2H), 1.89 (br, 1H), 1.60 (m, 2H), 1.37–1.23 (m, 6H), 0.88 (t, *J* = 6.8 Hz ,3H).

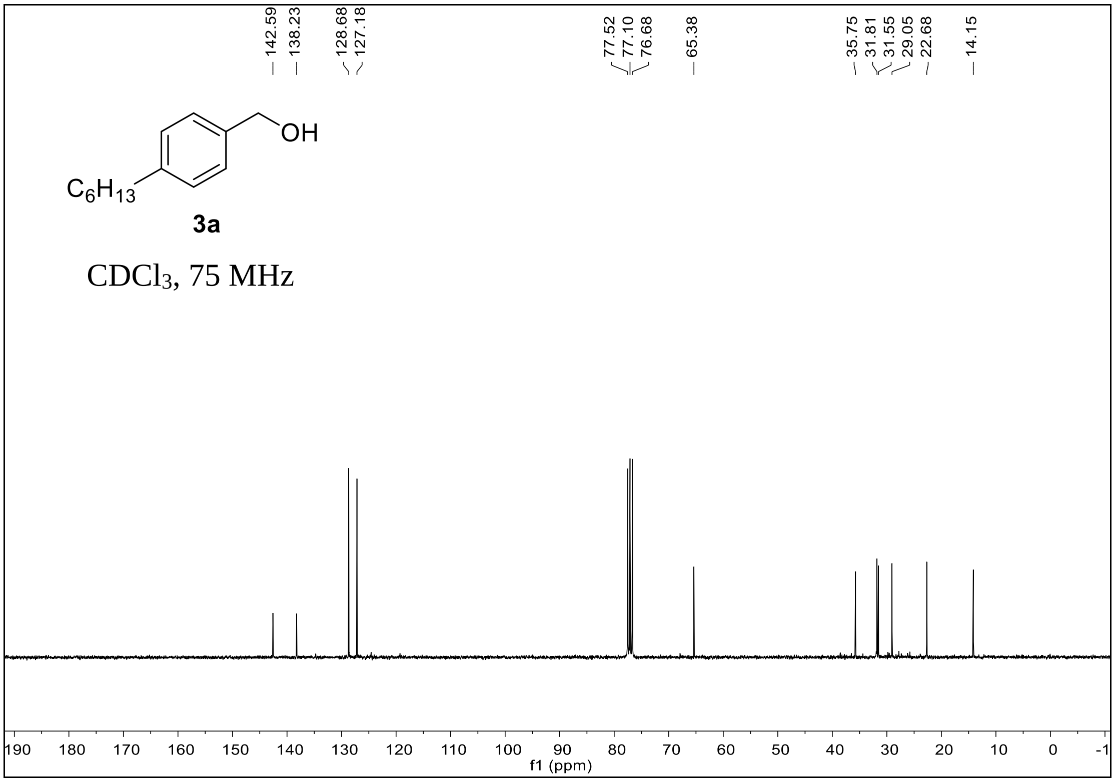

^{13}C NMR (75 MHz, $CDCl_3$) δ 142.6, 138.2, 128.7, 127.2, 65.4, 35.8, 31.8, 31.6, 29.1, 22.7, 14.2.

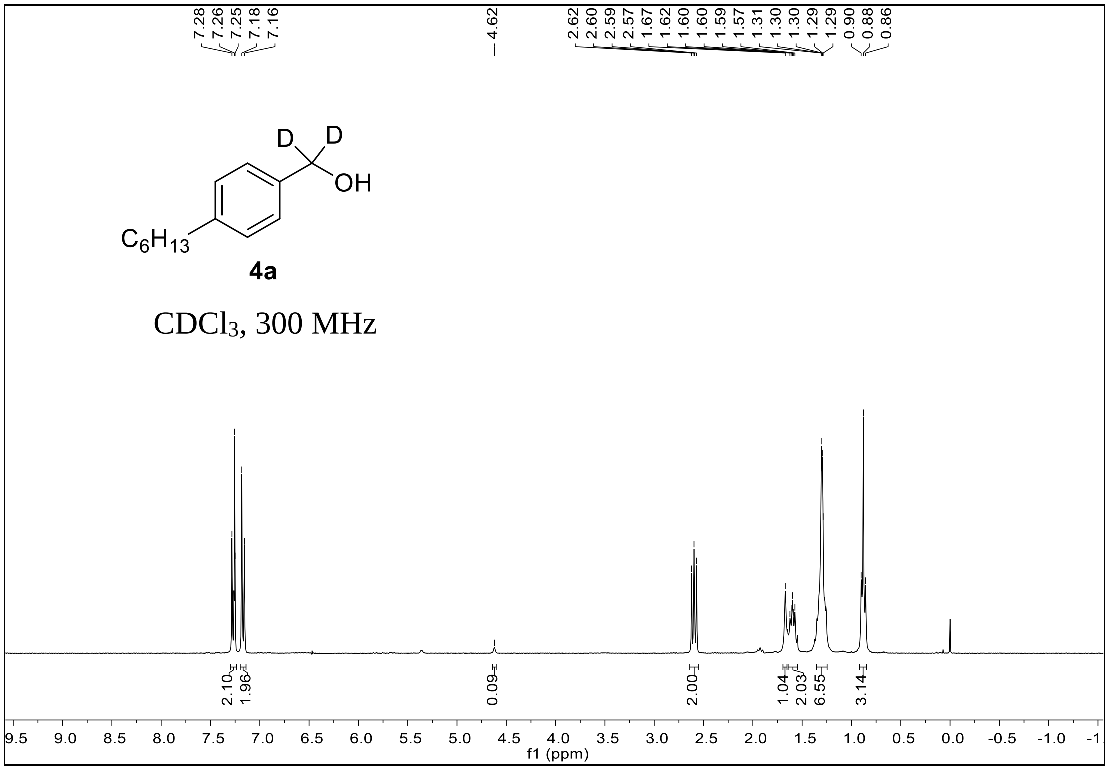

^{1}H NMR (300 MHz, $CDCl_3$) δ 7.26 (d, *J* = 8.1 Hz, 2H), 7.17 (d, *J* = 8.1 Hz, 2H), 2.60 (t, *J* = 7.7 Hz, 2H), 1.67 (br, 1H), 1.60 (m, 2H), 1.37–1.25 (m, 6H), 0.88 (t, *J* = 6.9 Hz, 3H).

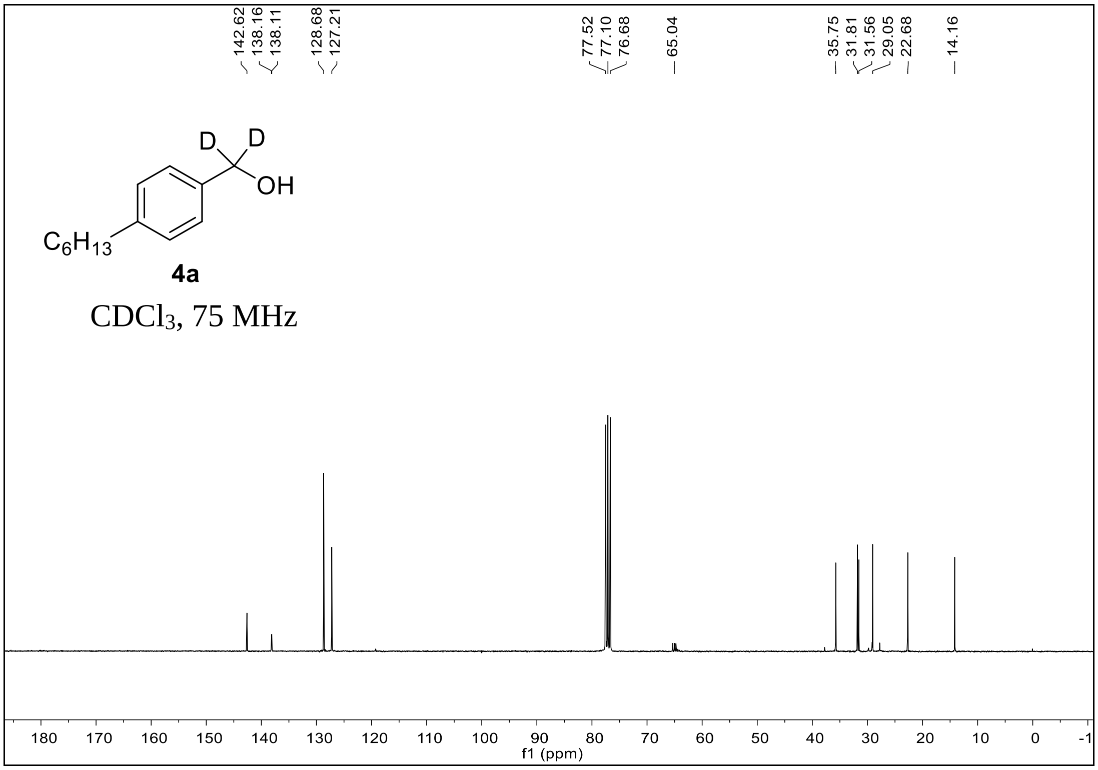

^{13}C NMR (75 MHz, $CDCl_3$) δ 142.6, 138.2 (m), 128.7, 127.2, 65.0 (m), 35.8, 31.8, 31.6, 29.1, 22.7, 14.2.

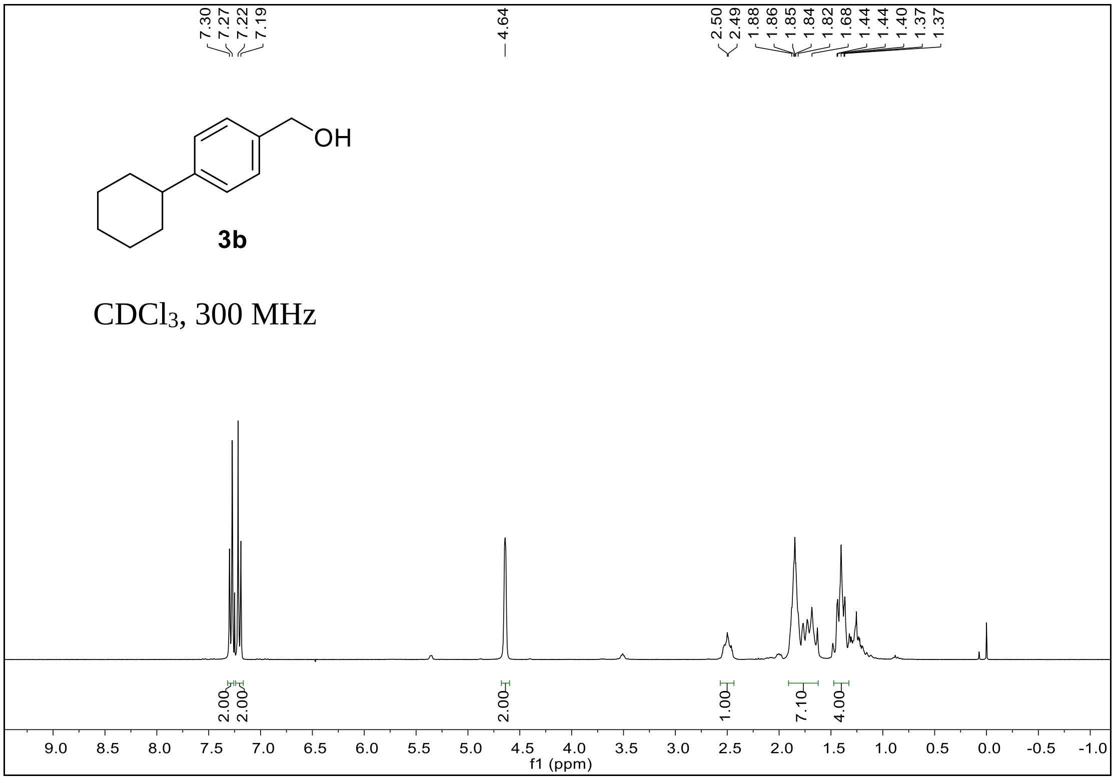

^{1}H NMR (300 MHz, $CDCl_3$) δ 7.29 (m, 2H), 7.20 (m, 2H), 4.64 (s, 2H), 2.49 (m, 1H), 1.93–1.60 (m, 7H), 1.49–1.33 (m, 4H).

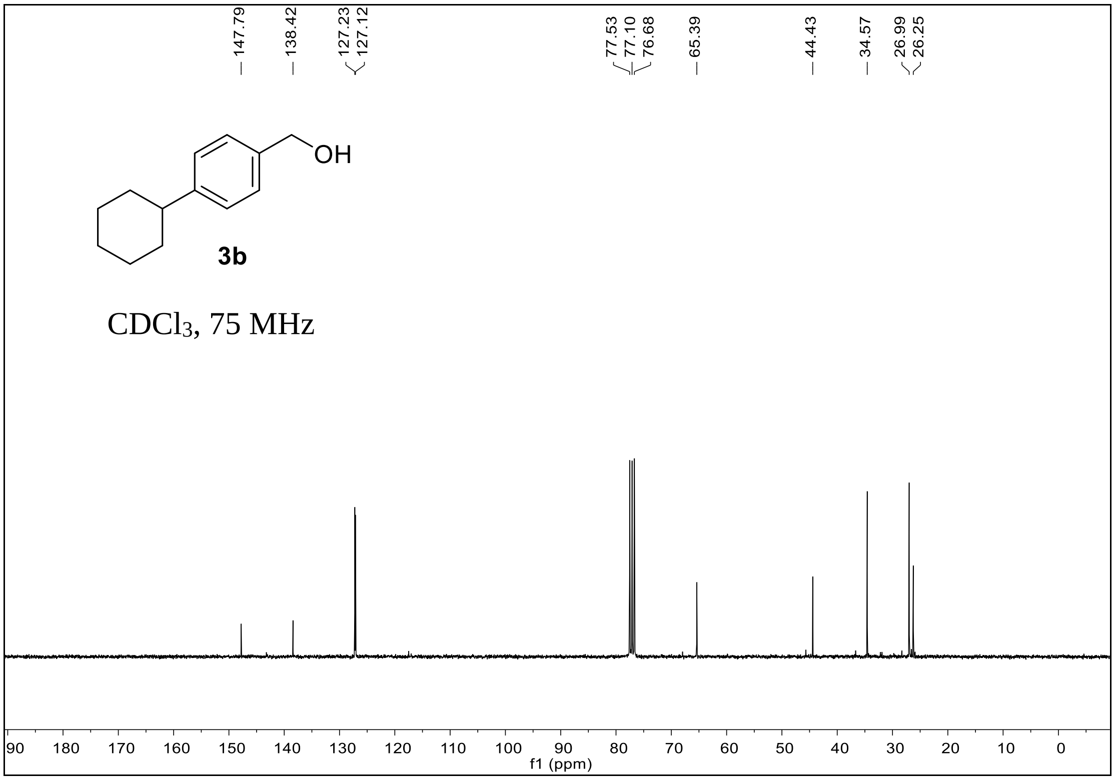

^{13}C NMR (75 MHz, $CDCl_3$) δ 147.8, 138.4, 127.2, 127.1, 65.4, 44.4, 34.6, 27.0, 26.3.

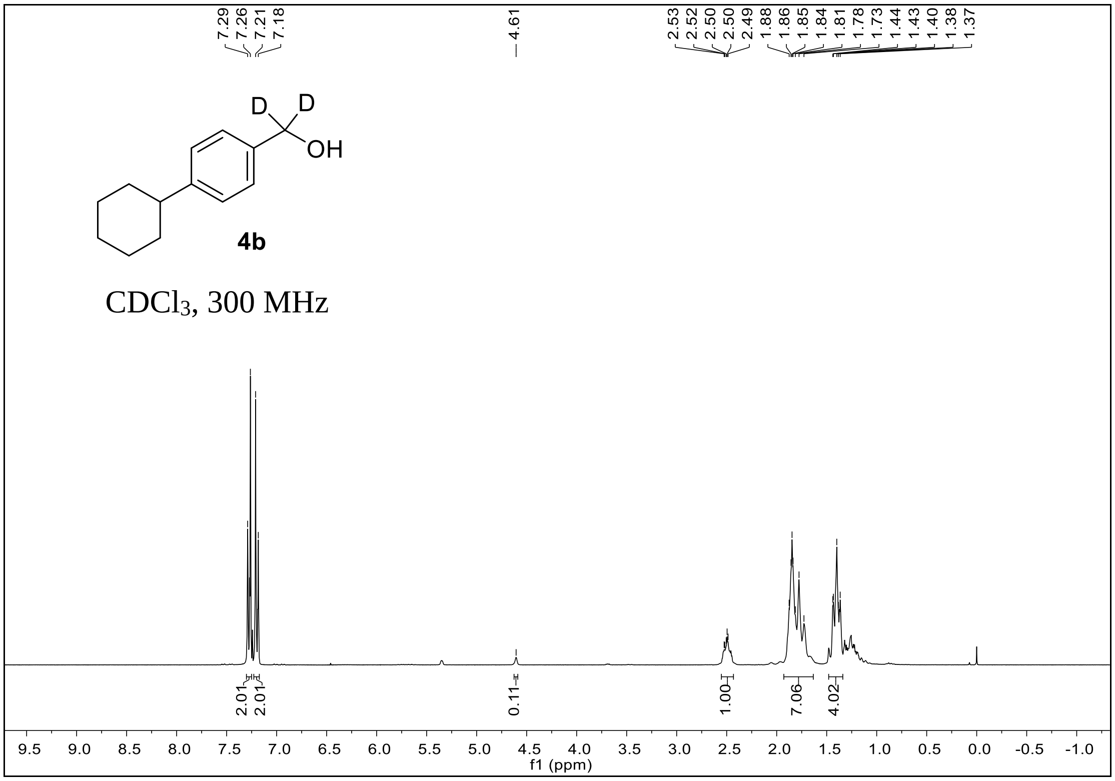

^{1}H NMR (300 MHz, $CDCl_3$) δ 7.28 (m, 2H), 7.20 (m, 2H), 2.50 (m, 1H), 1.93–1.64 (m, 7H), 1.48–1.33 (m, 4H).

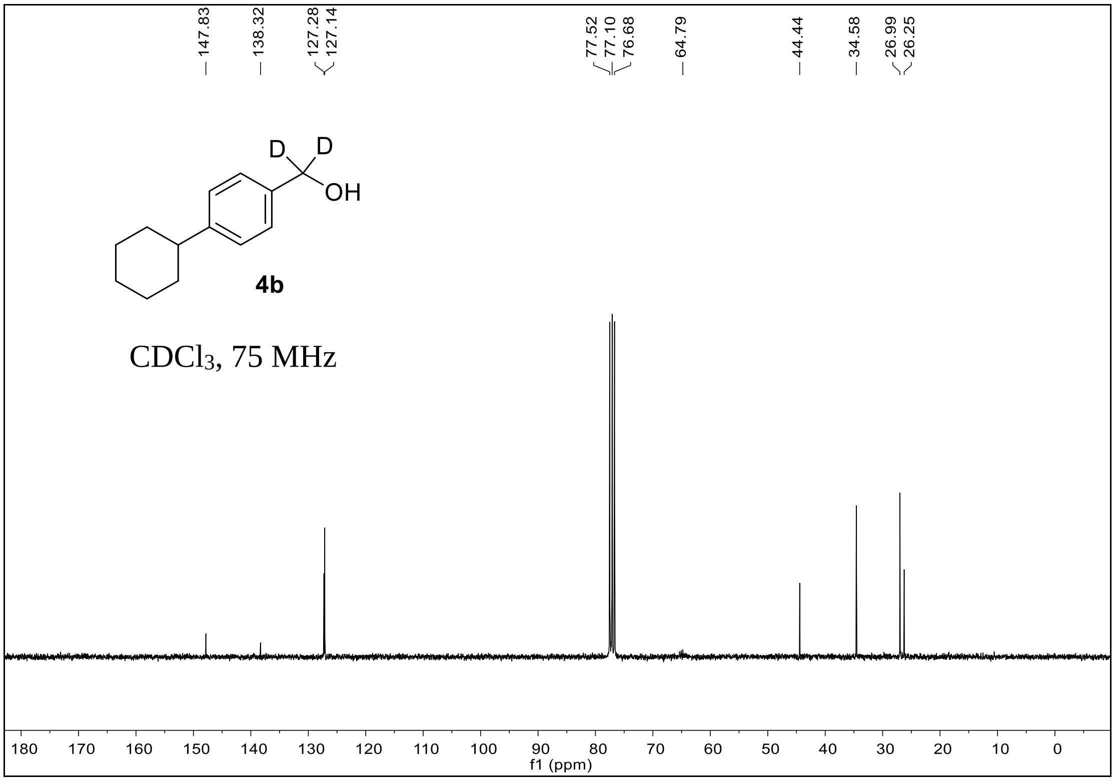

^{13}C NMR (75 MHz, $CDCl_3$) δ 147.8, 138.3, 127.3, 127.1, 64.8 (m), 44.4, 34.6, 27.0, 26.3.

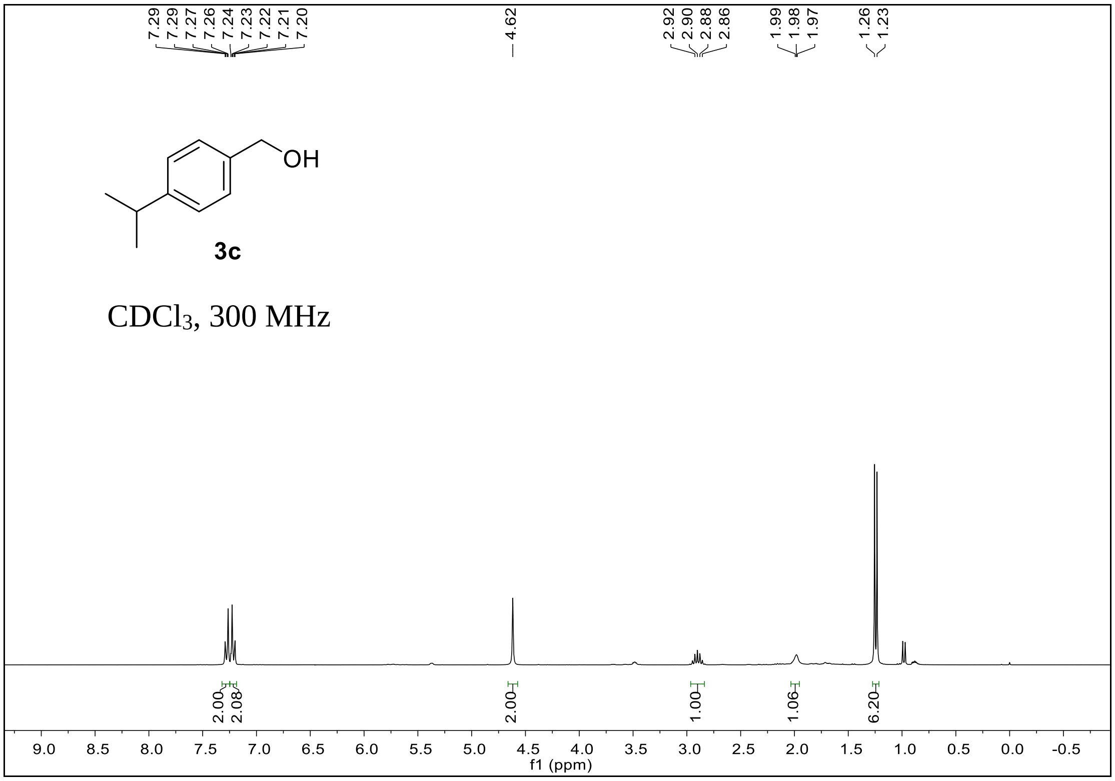

^{1}H NMR (300 MHz, $CDCl_3$) δ 7.29 (m, 2H), 7.22 (m, 2H), 4.62 (s, 2H), 2.90 (m, 1H), 1.98 (br, 1H), 1.24 (d, *J* = 6.9 Hz, 6H).

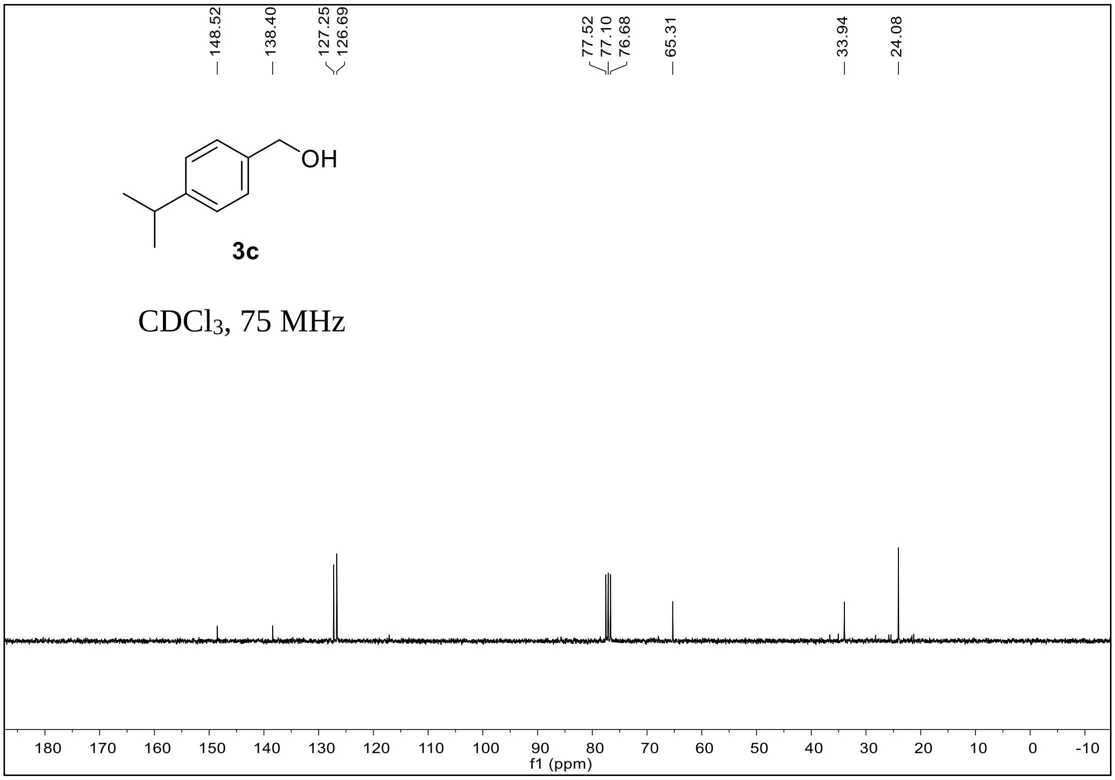

^{13}C NMR (75 MHz, $CDCl_3$) δ 148.5, 138.4, 127.3, 126.7, 65.3, 34.0, 24.1.

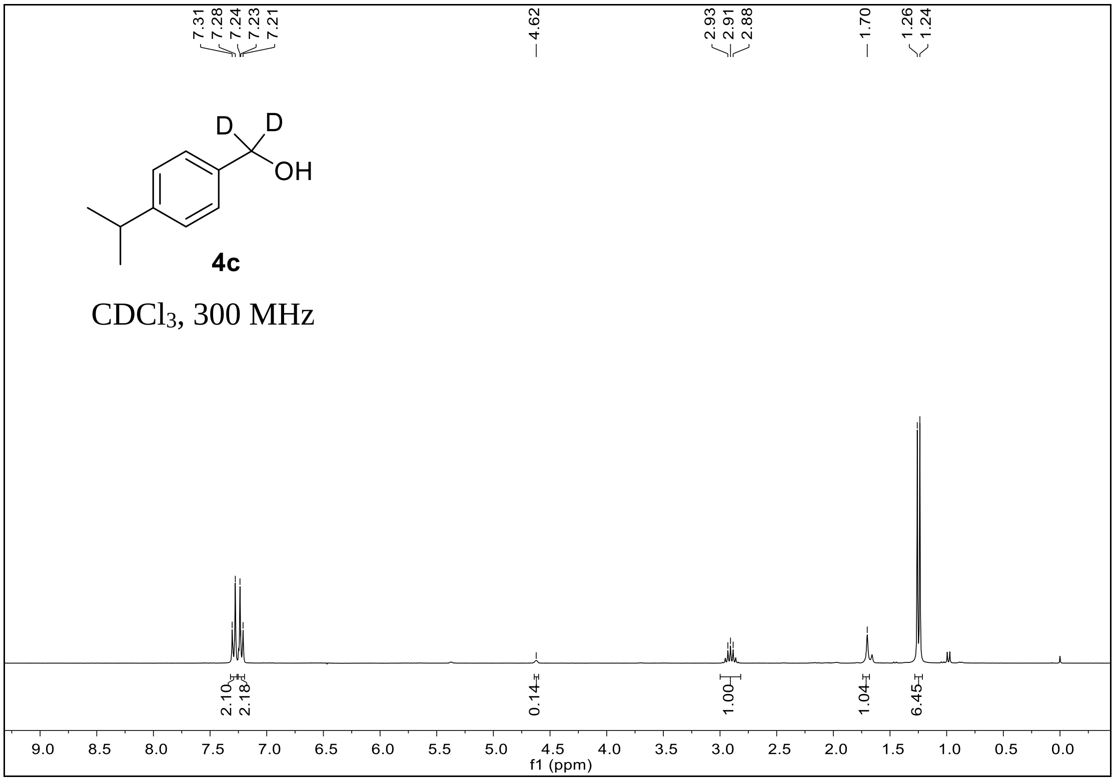

^{1}H NMR (300 MHz, $CDCl_3$) δ 7.29 (m, 2H), 7.22 (m, 2H), 2.91 (m, 1H), 1.70 (br, 1H), 1.25 (d, *J* = 7.0 Hz, 6H).

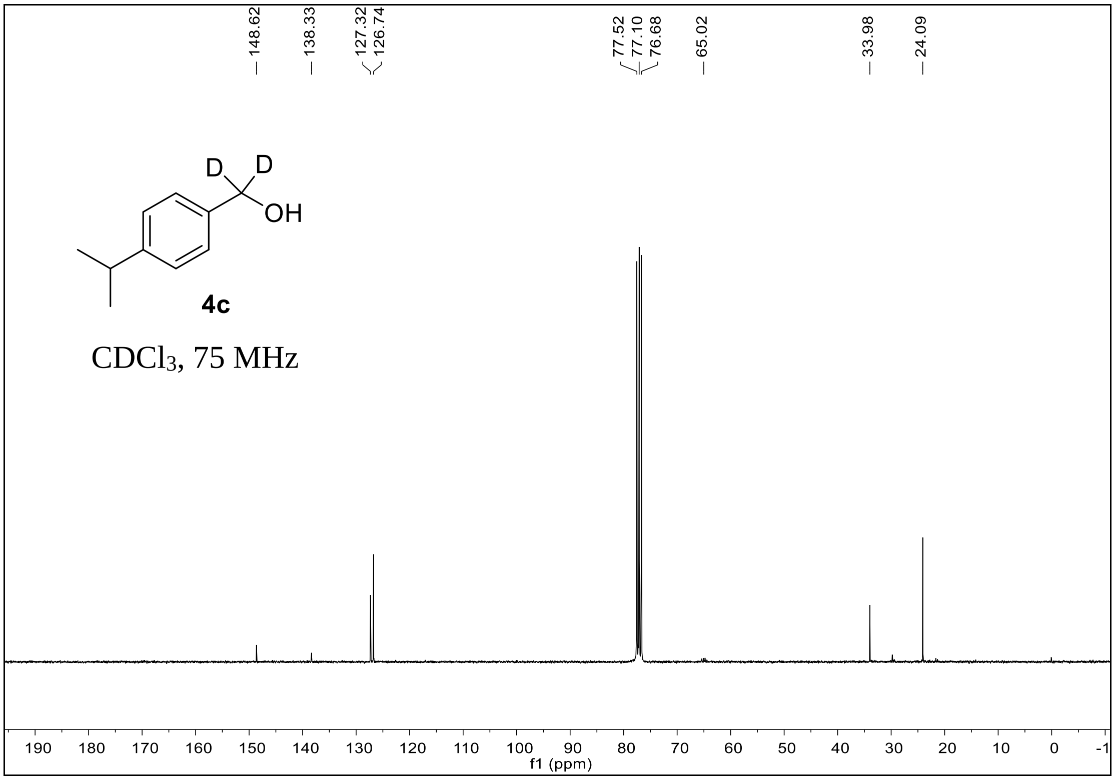

^{13}C NMR (75 MHz, $CDCl_3$) δ 148.6, 138.3, 127.3, 126.7, 65.0 (m), 34.0, 24.1.

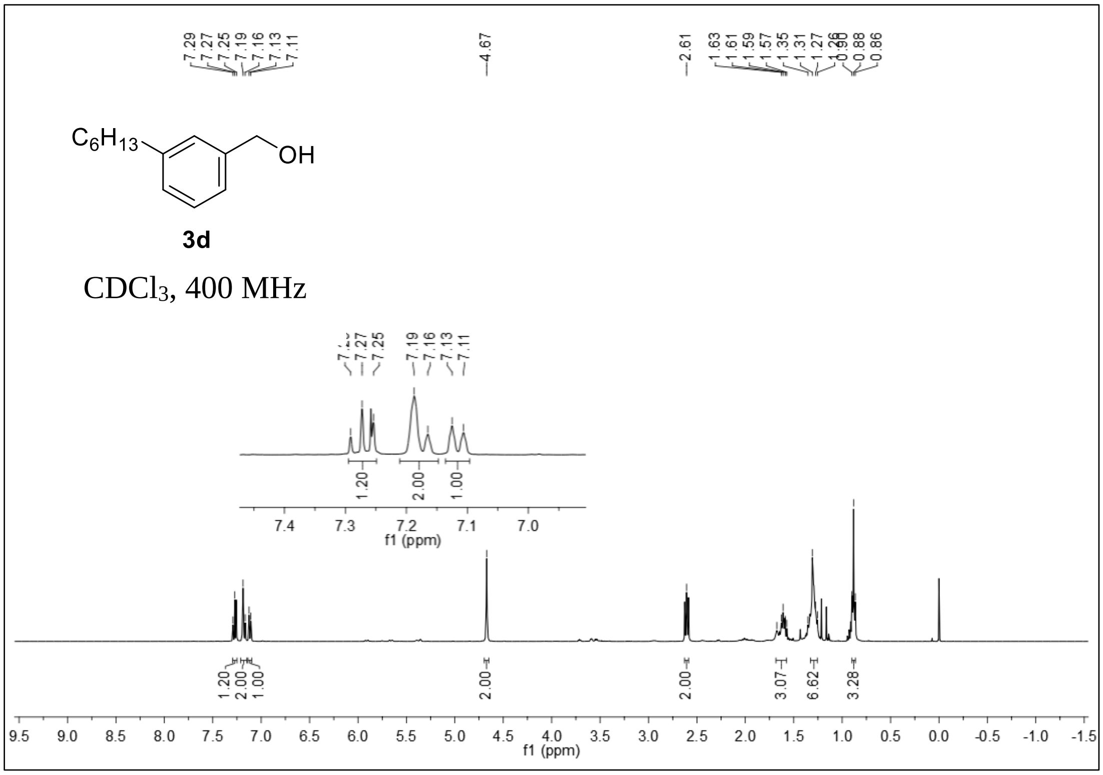

^{1}H NMR (400 MHz, $CDCl_3$) δ 7.30–7.25 (m, 1H), 7.21–7.15 (m, 2H), 7.14–7.10 (m, 2H), 4.67 (s, 2H), 2.61 (t, *J* = 7.7 Hz, 2H), 1.69–1.57 (m, 3H), 1.35–1.25 (m, 6H), 0.88 (t, *J* = 6.8 Hz, 3H).

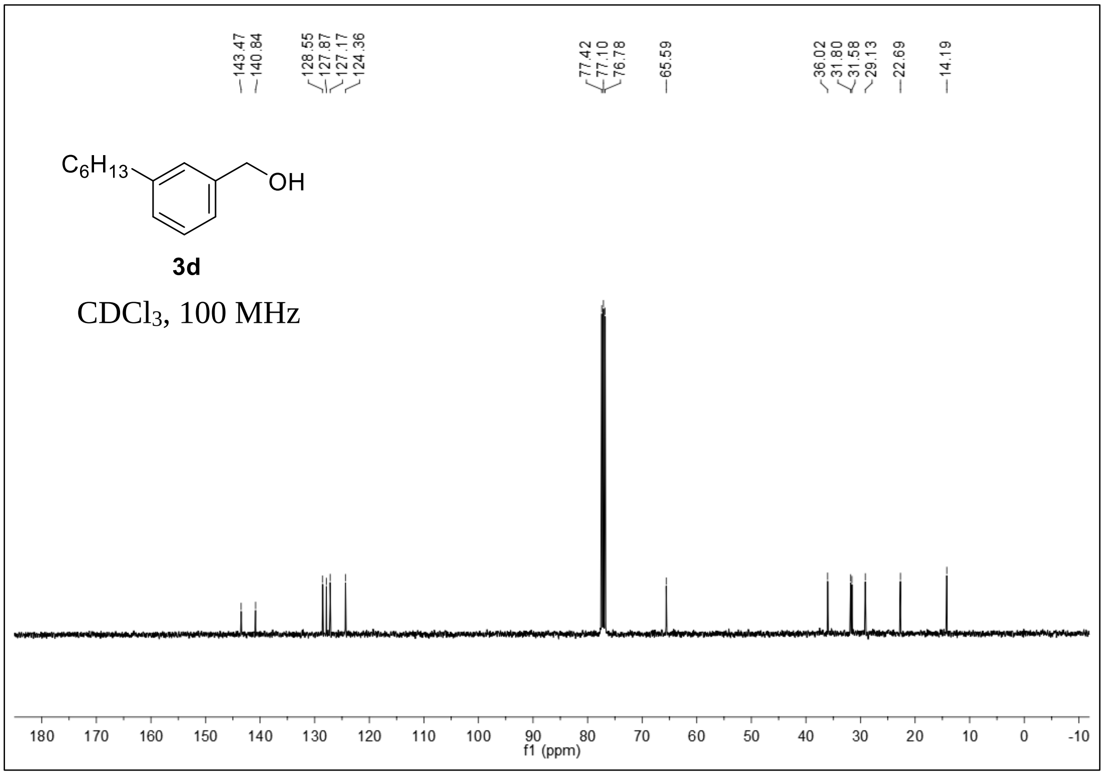

^{13}C NMR (100 MHz, $CDCl_3$) δ 143.47, 140.84, 128.55, 127.87, 127.17, 124.36, 77.42, 77.10, 76.78, 65.59, 36.02, 31.80, 31.58, 29.13, 22.69, 14.19.

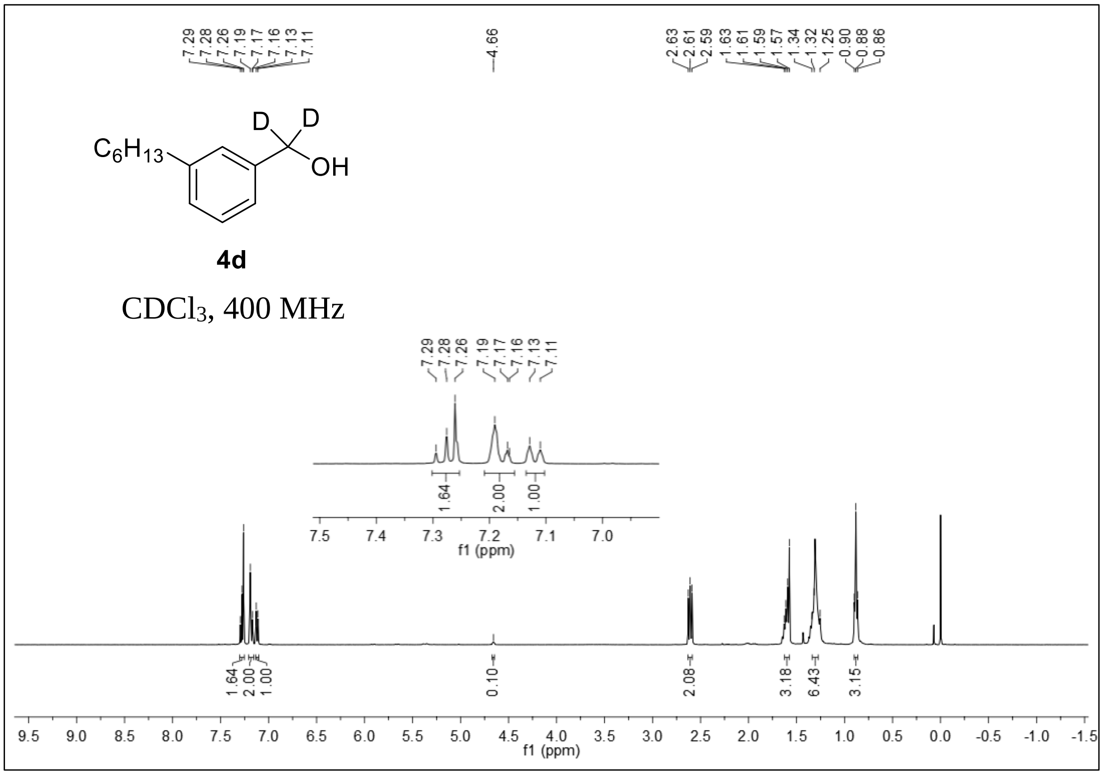

^{1}H NMR (400 MHz, $CDCl_3$) δ 7.30–7.25 (m, 1H), 7.21–7.15 (m, 2H), 7.14–7.10 (m, 2H), 2.61 (t, *J* = 7.7 Hz, 2H), 1.67–1.56 (m, 3H), 1.35–1.25 (m, 6H), 0.88 (t, *J* = 6.8 Hz, 3H).

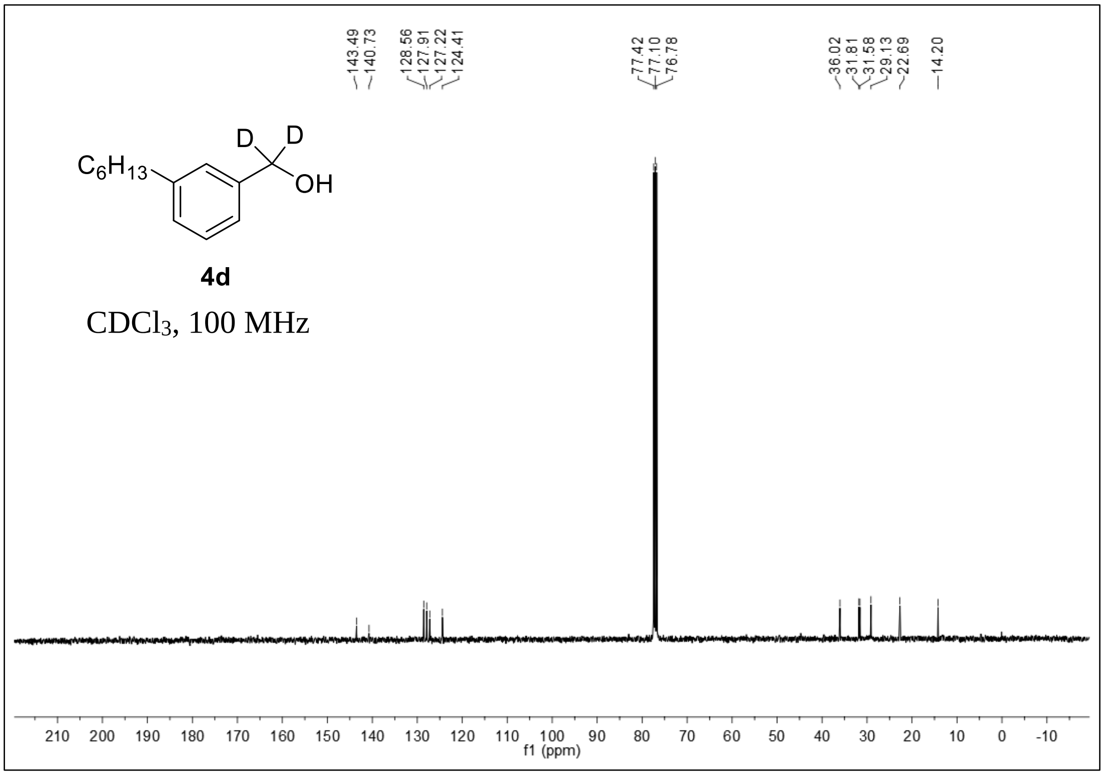

^{13}C NMR (100 MHz, $CDCl_3$) δ 143.49, 140.73, 128.56, 127.91, 127.22, 124.41, 77.42, 77.10, 76.78, 36.02, 31.81, 31.58, 29.13, 22.69, 14.20.
